# Halide Perovskite: A Promising Candidate for Next‐Generation X‐Ray Detectors

**DOI:** 10.1002/advs.202205536

**Published:** 2022-12-01

**Authors:** Ya Wu, Jiangshan Feng, Zhou Yang, Yucheng Liu, Shengzhong (Frank) Liu

**Affiliations:** ^1^ College of Chemistry and Chemical Engineering Xi'an Shiyou University Xi'an 710065 China; ^2^ Key Laboratory of Applied Surface and Colloid Chemistry National Ministry of Education Shaanxi Engineering Lab for Advanced Energy Technology School of Materials Science and Engineering Shaanxi Normal University Xi'an 710119 China; ^3^ State Key Laboratory of Catalysis Dalian National Laboratory for Clean Energy Dalian Institute of Chemical Physics Chinese Academy of Sciences Dalian 116023 China

**Keywords:** detectors, halide, imaging, perovskites, X‐ray

## Abstract

In the past decade, metal halide perovskite (HP) has become a superstar semiconductor material due to its great application potential in the photovoltaic and photoelectric fields. In fact, HP initially attracted worldwide attention because of its excellent photovoltaic efficiency. However, HP and its derivatives also show great promise in X‐ray detection due to their strong X‐ray absorption, high bulk resistivity, suitable optical bandgap, and compatibility with integrated circuits. In this review, the basic working principles and modes of both the direct‐type and the indirect‐type X‐ray detectors are first summarized before discussing the applicability of HP for these two types of detection based on the pros and cons of different perovskites. Furthermore, the authors expand their view to different preparation methods developed for HP including single crystals and polycrystalline materials. Upon systematically analyzing their potential for X‐ray detection and photoelectronic characteristics on the basis of different structures and dimensions (0D, 2D, and 3D), recent progress of HPs (mainly polycrystalline) applied to flexible X‐ray detection are reviewed, and their practicability and feasibility are discussed. Finally, by reviewing the current research on HP‐based X‐ray detection, the challenges in this field are identified, and the main directions and prospects of future research are suggested.

## Graphical abstract

The excellent photoelectric performance, simple preparation process and extremely low preparation cost of halide perovskites have not only attracte the attention of researchers but also obtain the favor from industry. This review summarizes the latest progress of perovskites for X‐ray detection in the past ten years, identify the challenges and suggest the main research directions in future.

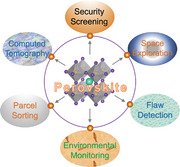

## 1 Introduction

Halide perovskite (HP) is a new semiconductor family with great potential in various optoelectronic applications.^[^ ^1^, ^2^ ^]^ HP materials have attracted much attention due to their excellent photoelectric properties, high carrier mobility, long carrier diffusion length, high color purity, tunable energy band, and high tolerance factor. These outstanding properties provide them with great potential in various photoelectric applications such as X‐ray detectors, solar cells, light transmission diodes and lasers. In the past decade, the power conversion efficiency (PCE) of perovskite solar cells (PSCs) has grown to 25.6%,^[^ ^3^ ^]^ which is very close to that of silicon solar cells. These remarkable developments depend on the quality of the perovskite film, which is due to the fabrication processes, the purity of materials, well‐matched charge transport layers, and appropriate solution formulation. However, the research progress of HP‐based detectors in terms of photoelectric performance and device application is still far behind those of PSCs and LED.^[^ ^4^ ^]^

**Figure 1:**
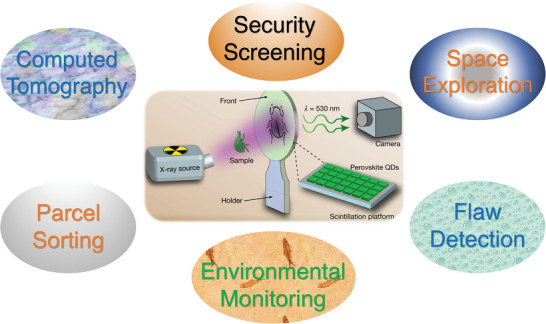
X‐ray detection application in different fields. Reproduced with permission.^[^ ^8^ ^]^ Copyright 2022, American Chemical Society.

In recent years, metal HPs have been demonstrated with high atomic number (Cs: 55, Pb: 82, Br: 35, and I: 53) constituents, adjustable energy band gap (*E*_g_ = 1.5–3.5 eV), high resistivity (*r* = 10^7^–10^12^ Ω cm), and large carrier mobility‐lifetime product (µ*τ* = 10^−5^–10^−2^ cm^2^ V^−1^), as well as low‐cost and low temperature single‐crystal growth.^[^ ^5^ ^]^ These superior characteristics endow perovskite single crystal and polycrystalline thin films with large radiation attenuation and improved carrier collection efficiency. Therefore, X‐ray detectors and imagers based on these perovskite materials have the characteristics of low detection limit, high stopping power, high spatial resolution, high sensitivity and energy resolution. In order to improve the efficiency and stability of X‐ray detection, the device configuration and structural dimension of high‐energy detectors has been explored at the molecular level. HP thin films with small grain sizes are used to confine the charge carries to improve radiative bimolecular recombination. HPs can absorb high‐energy X‐ray photons and convert them into low‐energy visible photons, so they could be widely used as detector materials in radiation exposure monitoring, safety inspection, non‐destructive testing, space exploration, package sorting, and medical imaging of radiography and computed tomography (CT) (**Figure** **1**).^[^ ^6^, ^7^ ^]^ However, the development of HPs for X‐ray detectors is still in its infancy. The inherent photoelectric properties of the materials, how the device structures affect their photoelectric properties, and some other corresponding problems have not been explored until now. Therefore, from the perspective of device physics, the commercialization of high‐performance X‐ray detectors still facing some challenges.^[^ ^4^ ^]^

This review summarizes the research progress of different types of perovskite X‐ray detectors in recent years, introduces the photoelectric characteristics and the basic physical parameters of carrier generation under different X‐ray detection modes, including direct and indirect X‐ray detection, and focuses on the detection mechanism of X‐ray detectors. Crystal structures and compositions, synthesis methods, morphological evolution and spectral response characteristics are discussed according to different X‐ray detector configurations. In addition, the research progress in improving the optical and mechanical properties of flexible X‐ray detectors is also reviewed. Finally, we briefly introduce the challenges faced by HP‐based X‐ray detectors, and look forward to their further application prospects.

## 2 Mechanism of X‐Ray Detection

### 2.1 Energy Conversion and Photoelectric Processes in X‐Ray Detection

**Figure 2:**
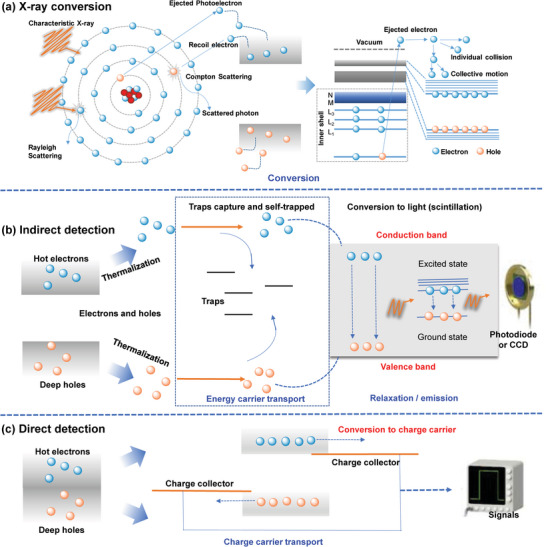
Energy conversion and photoelectric processes of X‐ray detection. a) Interactions between X‐ray photons and semiconductor: photoelectric effect, Compton scattering, Rayleigh scattering, and electron–hole pair generation. Reproduced with permission.^[^ ^9^ ^]^ Copyright 2022, Elsevier. b) The detection mechanism of indirect‐type X‐ray detector. Reproduced with permission.^[^ ^1^ ^]^ Copyright 2021, Wiley‐VCH. c) The detection mechanism of direct‐type X‐ray detector. Reproduced with permission.^[^ ^10^ ^]^ Copyright 2021, American Chemical Society.

For X‐ray detection, there are two strategies available. One is to use scintillators to indirectly convert X‐ray photons into low‐energy photons (indirect‐type), and the other is to use semiconductors to directly convert X‐ray photons into electronic signals (direct‐type). As for the X‐ray energy used for detection and scintillation, the interaction between X‐ray photons and matter mainly includes Rayleigh (coherent) scattering, photoelectric absorption, Compton (incoherent) scattering and electron–positron pair production, forming four interaction modes, as shown in **Figure** **2**a. The main detection mechanism of low‐energy photons (i.e., up to several hundred keV) depends on photoelectric absorption. Rayleigh (coherent) scattering is the slight scattering of bound atomic electrons by a photon interacting with the entire absorbing atom. Compton scattering is dominant at energies above several hundred keV. In the Compton scattering process, the photon loses part of its energy to the electron (recoil electron) and changes its path. In this process, photons are completely absorbed by atoms, and a photoelectron is ejected. When the photon energy exceeds 1.02 MeV, that is, 2 *m*_e_ *c* ^2^ (*m*_e_ is the static mass of the electron and *c* is the speed of light), an electron–positron pair will be generated.^[^ ^1^ ^]^ The generation of electron‐positron pair relies on the Coulomb field of atomic nuclei or orbital electrons. In addition, high‐Z materials are advantageous for the formation of electron‐positron pairs. In the photoelectric effect and Compton scattering, high‐energy secondary electrons, such as photoelectrons, Auger electrons and recoil electrons are generated by photons interacting with the semiconductor atom. The ejected electrons pass through the semiconductor and release many low‐energy free electrons through ionization. The free electrons rise to the conduction band (CB) and leave holes in the valence band (VB). The holes migrate to the top of the VB, and the free electrons lifted to the CB relax to the bottom of the CB through individual collisions or collective motion.^[^ ^1^ ^]^ Therefore, X‐rays generate electron–hole pairs in semiconducting material, which can be recombined to emit light or collected under an electric field to output current signal.

In Figure 2b, the X‐ray‐induced electron–hole pair emits photons in the scintillator through radiation recombination, which is part of the process of an indirect‐type detector.^[^ ^9^ ^]^ On the X‐ray plate, photons are further detected by p‐i‐n photodiodes or photomultiplier tubes and converted into charge signals. In direct‐type detectors (Figure 2c), X‐ray photons are absorbed by semiconductors and generate charge clouds in the body of the device. Defect states can capture electrons (holes) that are excited in the semiconductors. Before recombination, the holes may traverse between the electrodes many times, resulting in gain. The collected electrons and holes need to be stored and processed into electronic signals, and the pixel electrode collects electric charges and stores them in a storage capacitor, after that, the charge is transferred from the storage capacitor to the charge amplifier and converted into a voltage signal, which is quickly processed by the readout integrated circuit.^[^ ^10^ ^]^

The detection performance and applicability of direct‐type and indirect‐type X‐ray detectors are different in their detection mechanisms. For direct X‐ray detection, as there is only one time for the X‐ray photons to convert to electric signal, the energy loss is lower, which makes the detection signal easier and faster collected and then the signal reproducibility is better. Therefore, direct X‐ray detectors have advantages in high‐resolution applications due to their high sensitivity, high energy resolution, and better spatial resolution for X‐ray imaging. As for the indirect‐type X‐ray detection, the X‐ray photon needs to undergo two energy conversion processes and then output electrical signal, so the energy loss is more. In addition, because of the optical crosstalk during scintillation, the detection sensitivity and spatial resolution are suffered. Even so, the indirect‐type X‐ray detectors based on scintillators are mainstream products for X‐ray detection and imaging equipment in the market because of their fast response time and easy integration with the thin film transistor (TFT) and complementary metal oxide semiconductor (CMOS) photodetector arrays. Especially in portable X‐ray detection equipment, indirect detectors have more advantages, particularly in cost over the direct X‐ray detectors. However, the perovskite X‐ray detectors show promises to overturn the present status quo. According to the results reported so far, the direct‐type perovskite X‐ray detector can not only maintain high detection performance, but also greatly reduces its fabrication cost. Therefore, once the technology of perovskite X‐ray detector is getting more mature, it is expected that it will show advantages for direct‐type X‐ray detectors.

### 2.2 Direct‐Type X‐Ray Detectors

#### 2.2.1 Mechanism of Direct X‐Ray Detection

**Figure 3:**
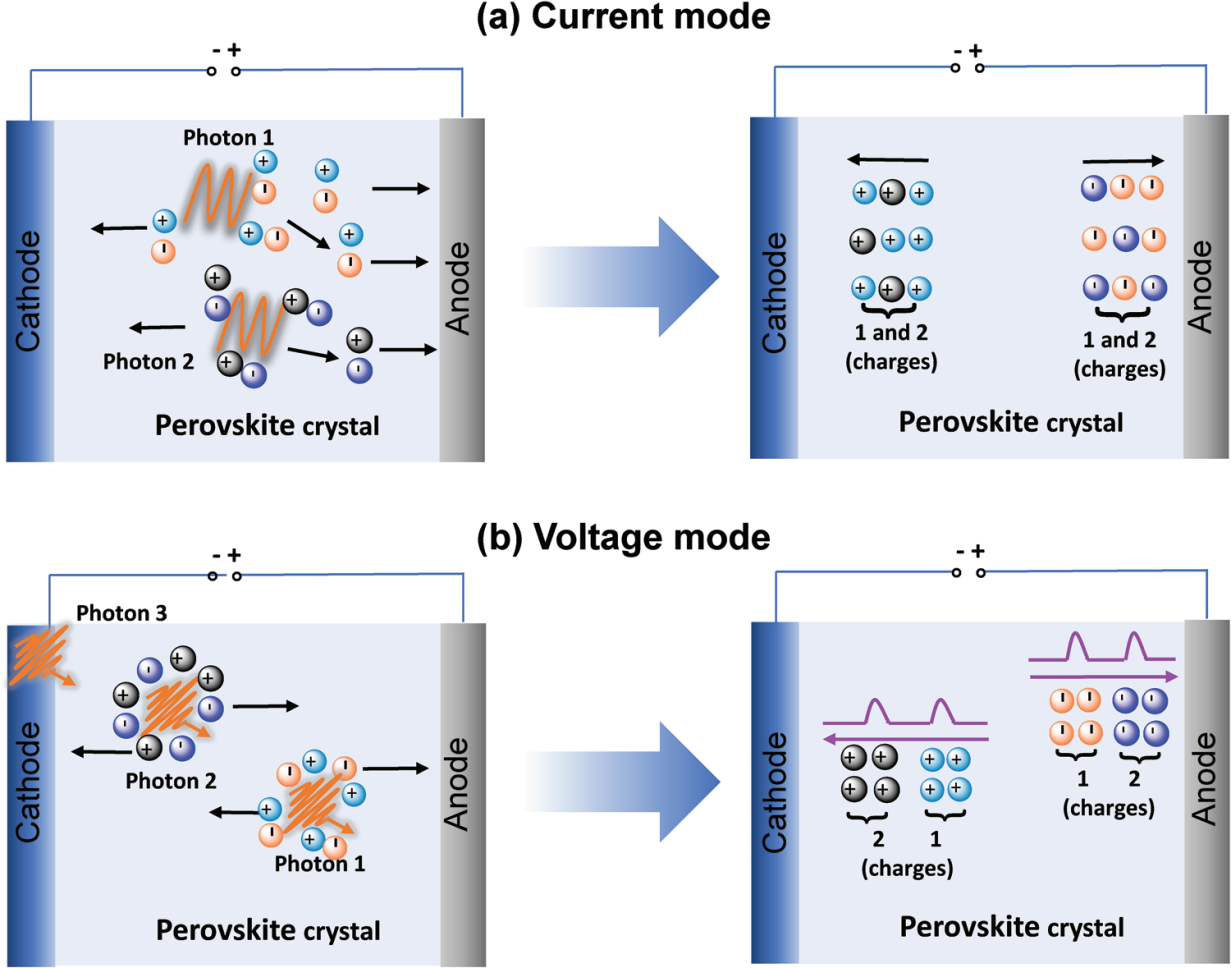
Operation mechanism of direct conversion X‐ray detectors. a) Current‐mode detector and b) pulse‐mode detector. Reproduced with permission.^[^ ^15^ ^]^ Copyright 2019, Springer Nature.

Direct‐type X‐ray detectors can be divided into current‐mode and voltage‐mode devices according to their working principles.^[^ ^9^, ^11^ ^]^ Current‐mode detection usually works with high light flux. The strong photon flux provides the X‐ray response with sufficient signal‐to‐noise ratio, thus leading to fast frame rate and smooth images.^[^ ^9^ ^]^ As shown in **Figure** **3**a, the current‐mode detector gathers high‐throughput and high‐energy photons, and then generates vast amounts of free charges through Compton scattering and other photoelectric effects. After that, the electrodes collect charge and generates a current signal under a certain bias. In the process, all X‐ray photons contribute to the transmission photon flux per unit time and contribute to the average value of signal current.^[^ ^12^ ^]^ Therefore, the current‐mode detectors can achieve sufficient signal‐to‐noise ratio and smooth image acquisition as well as excellent spatial resolution, so they are extensively used in irradiation imaging of materials and computed tomography.^[^ ^9^, ^13^, ^14^, ^15^ ^]^ In contrast, in the voltage‐mode, high‐energy photons are detected one after another, and thus the photon throughput intensity is quite weak, as shown in Figure 3b. Under a certain applied bias voltage each photon will generate some electron‐hole pairs, which are shifted to the opposite electrode, generating a temporary current. The peak intensity of the output current signal is proportional to the total induced charges. To integrate the weak transient current and convert it into a voltage pulse requires a charge‐sensitive preamplifier. The X‐ray photon energy spectrum is drawn according to the amplified voltage signals.^[^ ^16^, ^17^, ^18^ ^]^ Therefore, voltage‐mode (also called pulse‐mode) detection is usually suitable for photon‐deficient or low‐flux applications because capturing and forming electrical signals requires a long time. Pulse‐mode detectors are usually applied in spectroscopy, photon counters, and nuclear particle reaction monitors.^[^ ^15^ ^]^

Based on the discussion above, the current mode is often used to detect high‐throughput and high‐energy photons, which enables the detectors show strong current signal and therefore can realize high spatial resolution in X‐ray imaging. In addition, X‐ray detection with current mode needs lower requirements of the electronic environment when comparing with the pulse‐mode X‐ray detection, because its large enough signal‐to‐noise ratio makes it capable to process strong resistant to electromagnetic interference. In contrast, detectors operating at pulse‐mode are highly susceptible to interference from the external environment, so the equipment used for signal acquisition requires extremely high reading capability and resolution. Additionally, semiconductors used for single‐photon detection should to be sufficiently low defects and weak ion migration. However, due to the single‐photon detection capability of pulse‐mode, the detectors show high energy resolution.

#### 2.2.2 Key Parameters in the Performance of Direct‐Type X‐Ray Detector

Certain parameters are very important for evaluating direct X‐ray detectors. Although some applications require different characteristics, the key performance parameters of X‐ray detectors are sensitivity, charge collection efficiency, µ*τ* product, dark current, detection limit, and response speed. Some of these parameters are briefly described in following section.

##### Sensitivity

Sensitivity is a key parameter for direct‐type X‐ray detection that characterizes the ability of the detector to respond to a specific irradiation quantity.^[^ ^19^, ^20^ ^]^ High sensitivity indicates that X‐rays can induce a large photocurrent for a given irradiation dose rate, and it has a close relationship with the imaging quality. Generally, sensitivity (*S*) is determined by the collected charge per unit area under irradiation, which can be calculated by^[^ ^8^, ^21^ ^]^

(1) $$S=\frac{I_{p}-I_{d}}{D\times A}$$

where *I*_p_ and *I*_d_ are the photocurrent and the dark current, respectively, while *D* is the X‐ray dose rate, and *A* is the effective detection area. Therefore, one approach to improve the sensitivity is to increase the applied electric field, which can increase carrier separation and reduce recombination (increasing *I*_p_). However, a high electric field always produces a larger dark current and thus decreases the net X‐ray response current of the device. Therefore, the electric field shows important impact on the X‐ray detection sensitivity and thus a suitable electric field is necessary to achieve high sensitivity. Another one is to increase the carrier mobility of semiconductor, which enables the rapid transport of the X‐ray generated carriers and thus improve the carrier collection efficiency. Due to the larger carrier mobility, detectors based on HPs have achieved higher X‐ray detection sensitivity than the traditional semiconductor materials. Specifically, the highest sensitivity of HP X‐ray detector is exceeding 100 000 µC Gy_air_^−1^ cm^−2^.

##### Charge‐Collection Efficiency

The charge‐collection efficiency (CCE) of an X‐ray detector affects its sensitivity, therefore, the evaluation of a detector's CCE is indeed significantly.^[^ ^22^ ^]^ Generally, electron hole (E–H) recombination will happen at the interface because the carriers are captured and recombine there. The CCE of the detector can be calculated from the induced photocurrent (*I*_R_) and theoretical photocurrent (*I*_P_) with the following equation:^[^ ^23^, ^24^ ^]^

(2) $$\mathrm{CCE}=\frac{I_{R}}{I_{P}}$$

There are usually two strategies for improving the CCE. The first is to avoid charge trapping while increasing the drift length of carriers by utilizing a high bias. The second is to improve the quality of the perovskite material (reduce the trap density). Generally, the CCE decreases with increasing dose rate because carriers tend to localize in shallower traps, resulting in smaller gain.^[^ ^25^, ^26^ ^]^ For lead‐based HP materials, a large CCE is determined to exceeding 95% on the CsPbBr_3_ SC, which is comparable to CdZnTe and much bigger than a‐Se.^[^ ^27^ ^]^

##### µ*τ* Product

A large *µτ* product guarantees high transmission speed of X‐ray generated carriers and reduce their recombination due to aggregation, thus ensuring high high CCE under a low electric field. The parameter *µ* is the carrier mobility, that is, the drift velocity of carriers at 1 V cm^−1^, and *τ* is the carrier lifetime. The *µτ* product represents the distance that carriers drift in a unit electric field before annihilation, which can be derived from the modified Hecht equation:^[^ ^28^ ^]^

(3) $$I=\frac{I_{o}\mu \tau V}{L^{2}}\;\frac{1-\exp\left(-\frac{L^{2}}{\mu \tau V}\right)}{1+\frac{L}{V}\frac{s}{\mu }}$$

where *I*_o_ is the saturated photocurrent, *L* is the thickness, *V* is the applied bias, *τ* is the carrier lifetime. It is well known that the larger the *µτ* product, the higher the CCE.^[^ ^29^, ^30^, ^31^ ^]^ Also, high *µτ* product allows the detector to achieve high CCE at a low electric field. For perovskites, which belong to ionic semiconductors, the device will have better operating stability at lower electric field. The *µτ* product of HPs is generally larger than 10^3^ cm^2^ V^−1^, so the sensitivity of perovskite X‐ray detector is much higher than that of other semiconductor materials, making HPs a competitive material for next generation X‐ray detection.

##### Dark Current

The dark current, also known as the leakage current, is a significant parameter of a direct‐type X‐ray detector, and it has strict requirements for X‐ray planar pixel detectors. Three terms need to be considered. The first is that the high dark current fills the charge‐storage capacity and causes the device to quickly reach the TFT breakdown voltage; the size of the breakdown voltage is subject to the design of the TFT and is ≈40 V.^[^ ^1^, ^32^, ^33^ ^]^ The second is that a large dark current will increase the scattering noise, resulting in poor signal‐to‐noise ratio.^[^ ^34^ ^]^ The third is that the large dark current also cuts the dynamic range of the X‐ray planar pixel detector. A Schottky junction can be formed between the semiconductor and metal contact that prevents carrier injection. Kim et al. reduced the dark current of the device with a p‐i‐n structure.^[^ ^35^ ^]^ In addition, dark current drift is caused by ion migration. Since halide vacancies in the bulk and at grain boundaries are channels for ion migration, high‐quality SCs can reduce the dark‐current drift phenomenon. For polycrystalline films, increasing the crystallinity and grain size can reduce the amount of grain boundaries. In addition, grain‐boundary passivation can significantly weaken ion migration.^[^ ^36^ ^]^

##### Detection Limit

The detection limit quantifies the minimum X‐ray dose rate that can be reliably recognized by an X‐ray detector and is defined as the equivalent dose rate that produces a response signal three times larger than the noise level. The key to achieving a relatively low detection limit is to achieve a high current signal at a low noise level.^[^ ^37^, ^38^ ^]^ Besides, low dark current is also an important prerequisite to achieve low detection limit, because under the same level of X‐ray response, the lower dark current is conducive to the detector to obtain higher signal noise ratio (SNR) even at low dose rate. In addition to the dark current, low ion mobility in semiconductor also helps the detector to achieve a stable X‐ray response, further improving the SNR. As discussed in the above section, the X‐ray detectors fabricated on metal halide perovskites have the advantage of low dark current due to the high bulk resistivity. Furthermore, many reported studies have shown that metal halide perovskite can effectively inhibit internal ionic migration through composition engineering, thus achieving low detection limit.

##### Response Speed

Response speed refers to the time taken by the detector to respond to external stimuli. It is of great significance in X‐ray detectors for the reason that a fast response speed can shorten the exposure time and allow the imaging process to utilize a higher frame rate.^[^ ^39^ ^]^ Specifically, for medical diagnosis, a short response time is the key parameter for realizing dynamic real‐time X‐ray imaging. The response time is defined as the rise time of the pulse peak from 10% to 90% (rise time, *τ*_1_) and then from 90% to 10% (decay time, *τ*_2_) along with the required stable output time. Generally, it is closely related to the number, state and quality of the traps of the material.^[^ ^40^ ^]^

### 2.3 Indirect‐Type X‐Ray Detectors

#### 2.3.1 Mechanism of Indirect X‐Ray Detection

In the indirect detector, a scintillator or phosphor converts X‐rays with high photon energy into low‐energy ultraviolet/visible (UV/vis) photons, which can be further detected by array photodetectors (e.g., amorphous Si photodiodes and photomultipliers).^[^ ^41^ ^]^ The scintillation process of inorganic scintillators mainly includes three stages (conversion, transport, and luminescence). During the conversion phase, incident X‐rays (100 eV < *h*_*ν*_ < 100 keV) interact with the lattice atoms of the material and undergo thermal electron Compton scattering when the radiation energy range is less than 1 MeV. When the radiation energy is greater than 1.02 MeV, the generation of electron‐positron pairs also contributes to the generation of carriers. Through electron scattering and the Auger process, abundant secondary electrons are generated, resulting in the generation of carriers with low kinetic energy. In addition, the energy of these carriers is thermally dissipated by interaction with phonons.^[^ ^42^ ^]^ In this process, a large number of low‐kinetic‐energy electrons and holes gradually accumulate separately in the CB and the VB. It is worth noting that the whole conversion stage occurs on the sub‐picosecond time scale. Many electrons and holes then enter the transport stage of the luminescent center, which usually occurs in the time range of 10^−12^–10^−8^ s. In the transport stage, the migrated carriers may be trapped by defects in the scintillator (such as ion vacancies and grain boundaries) or self‐trapped in the lattice, resulting in non‐radiation loss and possible radiation recombination delay. Optimizing the crystal growth or surface morphology of the scintillator can effectively suppress defects. In the last stage, luminescence, the capture, and radiative recombination of electron–hole pairs produce X‐ray luminescence, which is luminescence in the UV–vis region. It is worth noting that the above scintillation process mechanism is mainly applicable to inorganic scintillators, but it is more complex for organic scintillators or amorphous scintillators.^[^ ^43^ ^]^

#### 2.3.2 Key Parameters in the Performance of Indirect‐Type X‐Ray Detector

Scintillators based indirect X‐ray detectors have the advantages of fast response time and convenient integration with TFT or CMOS arrays, etc., and they are the mainstream products in the market at present. The band gap of HPs can be tuned by adjusting the B/X site to match the photodetector response wavelength,^[^ ^44^ ^]^ leading to a rapid luminescence attenuation process (≈nanosecond). In addition, the photoluminescence quantum yield (PLQY) of HP nanocrystals is close to 100%, which can enable low‐afterglow X‐ray imaging. The charge readout process is the same as for the direct‐conversion process after photons are converted into charges, which has been discussed above. Unlike direct detection, the X‐ray‐induced electron‐hole pair emits photons in the scintillator through radiative recombination, as shown in Figure 2b. On the X‐ray plate, photons are further detected by p‐i‐n photodiodes and converted into signal charges. More details will be discussed in the following sections. Here, the key figure‐of‐merit parameters used to characterize the indirect X‐ray detector can be summarized as follows.

##### Afterglow

Afterglow is the intensity of radiation luminescence at a given time after X‐ray radiation. Different applications have different requirements for afterglow. The highest requirement for CT is 0.1% @ 3 ms. Since the signal is superimposed on the previous exposure, long afterglow will cause imaging artifacts.^[^ ^45^, ^46^, ^47^ ^]^ Compared with traditional scintillators, HPs show much short afterglow. For example, the afterglow of CsPbBr_3_ nanocrystal was determined to be about 1% @ 30 ns, which is much better than the commercialized terbium‐doped gadolinium oxysulfide (GOS)‐based indirect‐type detectors,^[^ ^48^ ^]^ and another all‐inorganic perovskite Cs_2_Ag_0.6_Na_0.4_In_0.85_Bi_0.15_Cl_6_ scintillator has been demonstrated with a afterglow of 0.1% @ 16 µs, which is also superior than the mainstream scintillator CsI:Tl (1.5% @ 3 ms).^[^ ^1^ ^]^ The theoretical analysis shows that the low‐dimensional structure HPs have the intrinsic quantum well structures, so the exciton binding energy can be adjusted by adjusting the dimension of HP to further short the afterglow to hundreds of picoseconds.^[^ ^49^ ^]^

##### Light Yield

Light yield (LY) is another important parameter of a scintillator that determines the sensitivity and detection limit of the detector. It is defined as the number of photons absorbed by the scintillator per 1 MeV of energy when exposed to ionizing radiation (usually one gamma photon). The number of emitted photons (*N*_ph_) generated by scintillation can be calculated by^[^ ^10^ ^]^

(4) $$N_{ph}=\frac{E}{\beta E_{gap}}\times \mathrm{SQ}$$

where *E* is the energy of the incident X‐ray photon, and *E*_gap_ is the band gap of the scintillator. *S* and *Q* are the quantum efficiencies of the transport stage and the luminescence stage, respectively, and *β* represents the average energy required to generate a thermalized electron–hole pair, which is a phenomenological parameter, usually between 2 and 3. Compared with other scintillators used for X‐ray detection, HP has a theoretical LY of ≈200 000 photons MeV^−1^ due to its defect tolerance and relatively small band gap.^[^ ^50^ ^]^ However, the actual LY value may be reduced due to self‐absorption, non‐radiative recombination, and nonuniform light extraction efficiency. Due to the self‐absorption effect, the generated photons can be reabsorbed by the scintillator itself, thereby reducing LY. In addition, defects in the scintillator lead to non‐radiative recombination, which further reduces LY. In practical applications, reflective coatings are used to avoid light loss, and some optical coupling adhesives are used between scintillators and photodiodes to ensure light extraction efficiency.^[^ ^51^ ^]^

##### Emission Wavelength

The emission wavelength of the scintillator also needs to be optimized to maximize LY. *α*‐Si‐based p‐i‐n photodiodes usually achieve the highest response in the wavelength range of 500–600 nm.^[^ ^52^ ^]^ When the emission wavelength of the scintillator matches the response peak of the photodiode, it is perfect. The emission wavelength of the HP can be continuously adjusted by adjusting the B/X site. Chen et al. adjusted the X site of CsPbX_3_ (X = Cl, Br, I) nanocrystals, and scintillators with different luminescence covers from UV to red were prepared.^[^ ^53^ ^]^

## 3 Advantage Properties of HPs for X‐Ray Detection

In recent years, HP has become a typical material for X‐ray detection and imaging due to its low cost, outstanding inherent electronic and optical characteristics, such as large X‐ray absorption coefficient, high PLQY, tunable band gap, low defect density, large mobility, and long carrier recombination life, even in the form of polycrystalline thin films prepared in solution. HP‐based X‐ray detection has developed rapidly due to its unique advantages such as low cost, outstanding inherent electronic and optical characteristics, easy integration with readout electronics, and better spatial resolution of X‐ray imaging. All these characteristics are actually required for X‐ray detection. These favorable properties of HPs are summarized and discussed in detail in the following sections.^[^ ^32^, ^54^ ^]^ In this section, we introduce the advantages of HPs, which make them a new generation of X‐ray detection materials.

### 3.1 Outstanding Electronic and Optical Properties

#### 3.1.1 Large Absorption Coefficient

**Figure 4:**
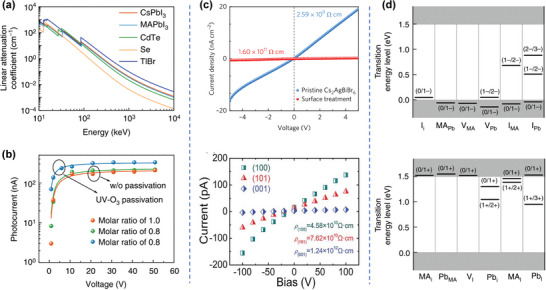
Electronic and optical properties of HPs. a) Linear attenuation coefficient of MAPbI_3_, MAPbBr_3_, CdTe, Se, and TlBr versus photon energy. b) The µ*τ* product of MAPbBr_3_ crystal devices with different feed ratios of raw materials and surface passivation procedures. Reproduced with permission.^[^ ^15^ ^]^ Copyright 2019, Springer Nature. c) Resistivity of Cs_2_AgBiBr_6_ and Cs_3_Bi_2_I_9_ single crystals. (top) Reproduced with permission.^[^ ^55^ ^]^ Copyright 2017, Springer Nature. (bottom) Reproduced with permission.^[^ ^56^ ^]^ Copyright 2017, Springer Nature. d) The transition energy levels of intrinsic acceptors and intrinsic donors in MAPbI_3_ perovskite. Reproduced with permission.^[^ ^15^ ^]^ Copyright 2019, Springer Nature.

The existence of the high‐Z ions in HPs provide them with good X‐ray absorption capacity. Cs, Pb, Bi, I, and Br are common elements in HPs. For example, the most‐studied MAPbBr_3_ and MAPbI_3_ (MA = CH_3_NH_3_^+^) HPs have linear absorption coefficients of 19.41 and 40.61 cm^−1^ for 50 keV X‐rays, respectively, (**Figure** **4**a) which are much higher than those of Si (1.02 cm^−1^) and *α*‐Se (3.86 cm^−1^).^[^ ^15^, ^57^ ^]^ Under the same conditions, the linear absorption coefficient of CsPbI_3_ reaches 57.06 cm^−1^, which is equivalent to that of CdZnTe (60.63 cm^−1^). In recent years, a large number of lead‐free HPs (such as Cs_2_AgBiBr_6_ and Cs_3_Bi_2_I_9_) have been applied to X‐ray detection and imaging applications. The heavier stabilizing element Bi allows more effective X‐ray attenuation than for lead‐based HPs.

#### 3.1.2 High µ*τ* Product and Large Resistivity

The semiconductor detectors generate electric charges by absorbing X‐rays. In order to collect these charges, the moving distance of electrons and holes should be much longer than the thickness of the semiconductor layer. Therefore, under a given electric field, the *µτ* product directly determines the CCE, which is an important parameter in determining the X‐ray sensitivity. In polycrystalline semiconductor thin films, the better the film quality, the larger the *µτ* product. HP has the characteristics of high mobility, long carrier recombination life and strong defect resistance. For example, the *µτ* product of MAPbI_3_ polycrystalline film is 2 × 10^−7^ cm^2^ V^−1^, and the *µτ* product of *α*‐Se is ≈10^−7^cm^2^ V^−1^, which is equivalent. Because single crystal (SC) material has high crystallinity and no grain boundaries, its defect density is much lower than that of polycrystalline film. In MAPbI_3_ SC, the *µτ* product reaches 1.2 × 10^−2^ cm^2^ V^−1^, generating a carrier diffusion length of 175 µm (Figure 4b).^[^ ^58^, ^59^ ^]^ Recently a *µτ* product up to 1.2 × 10^−1^ cm^2^ V^−1^ was reported for Cs_0.1_FA_0.9_PbBi_2.8_Br_0.2_ SC (FA = CH(NH_2_)_2_).^[^ ^60^, ^61^, ^62^ ^]^ This value is even comparable to those of CdZnTe and CdZnTeSe SCs at room temperature.

Operation at a high electric field can improve the X‐ray detection sensitivity. However, since high voltage bias usually generates a large dark current, it is necessary to consider the balance between limited sensitivity and noise‐limited sensitivity. In order to suppress the dark current, we should consider semiconductors with high resistivity. The bulk resistivity of lead HP is generally between 10^7^ and 10^10^ Ω cm. The resistivity of Cs_2_AgBiBr_6_ and Cs_3_Bi_2_I_9_ SCs can reach 1.6 × 10^11[^ ^55^ ^]^ and 1.24 × 10^12^ Ω cm (Figure 4c).^[^ ^56^ ^]^The large bulk resistivity leads to small dark current, which is conducive to low‐noise X‐ray detection.

#### 3.1.3 Radiation Hardness

The radiation hardness refers to the stability or repairability of the materials after being exposed to high dose or continuous radiation. Generally, materials with excellent radiation hardness tend to with a high radiation damage limit, or they can self‐repair after being damaged by radiation. A unique characteristic of HP is that it has a better tolerance for defects than other semiconductor materials. First‐principles calculations show that deep‐level defects in MAPbI_3_, such as I_Pb_, I_MA_, Pb_i_, and Pb_I_, have large formation energies (Figure 4d).^[^ ^63^ ^]^ Most of the point defects, whether in the conduction band or valence band, are shallow defects, such as MA_i_, V_Pb_, MA_Pb_, I_I_, V_I_, and V_MA_. The defect tolerance results in radiation damage tolerance.^[^ ^64^, ^65^, ^66^ ^]^ It is encouraging to find that, in one report, a device displayed self‐healing ability, that is, after 10 days of storage on the shelf, the current density of the device returned to the initial value.^[^ ^67^ ^]^ Chen et al. found that applying bias voltage to perovskite will produce Frenkel defects, which may be related to the loss of current density under irradiation because the built‐in electric field may also cause Frenkel defects.^[^ ^68^ ^]^ The self‐annihilation of the Frenkel defect was also confirmed when the electric field was released, which is consistent with the self‐healing effect observed. It has been demonstrated by Zhang et al., the radiation hardness of HP SC is comparable to that of the commercial CZT detector.^[^ ^69^ ^]^ Furthermore, the HP with low‐dimensional structure has extremely high radiation resistance. For example, the MA_3_Bi_2_I_9_ SC detector can be irradiated continuously for tens of hours under high electric field while shows only negligible changes in dark current and photocurrent. Which further demonstrated the extremely superior radiation hardness of HPs. More performance comparison of HPs X‐ray detectors and other semiconductor X‐ray detectors are summarized in **Table** **1**.

**Table 1:** Performance comparison of perovskite‐based and other semiconductor X‐ray detectors

Materials	µ*τ* product [cm^2^ V^−1^]	Electric field [V mm^−1^]	Sensitivity [µC Gy^−1^ cm^−2^] [kV]	Detection limit [nGy s^−1^]	Refs.
MAPbBr_3_ SC	4.0 × 10^−3^	≈467	21 000(8)	<100	[70]
MAPbBr_3_ SC	1.2 × 10^−2^	15	80(50)	≈500	[71]
MAPbBr_3_ SC	2.59 × 10^−2^	150	3928.3(120)	<8800	[72]
MAPbBr_3_ SC	4.1 × 10^−2^	≈8.3	259.9(39)	‐	[73]
MAPbBr_3_ SC	2.6 × 10^−4^	50	≈529	1210	[74]
MAPbBr_3_ SC		≈19.8	184.6	<1200	[75]
MAPbBr_2.94_Cl_0.06_ SC	1.8 × 10^−2^	600	84 000	7.6	[76]
MAPbBr_3_ SC arrays		≈300	242(100)	8500	[77]
MAPbBr_3_ Crystallines	‐	71	488(60)	2300	[78]
MAPbI_3_ film	2.0 × 10^−7^	‐	‐	‐	[79]
MAPbI_3_ film	1.0 × 10^−4^	2400	11 000(100)	‐	[35]
MAPbI_3_ SC	3.26 × 10^−3^	≈10	968.9	‐	[80]
MAPbI_3_ SC(//)	‐	100	≈7× 10^5^ (50)	1.5	[81]
MAPbI_3_ SC	‐	15	21 000(60)	18000	[82]
MAPbI_3_ Wafer	2.0 × 10^−4^	2000	2527(70)	48000	[83]
MAPbI_3_ Wafer	3.84 × 10^−4^	12.5	122 000 (40)	‐	[84]
MAPb(I_0.9_Cl_0.1_)_3_‐filled membrane	1.5 × 10^−3^	500	≈8696(60)	‐	[85]
Cs_0.1_(FA_0.83_MA_0.17_)_0.9_Pb(Br_0.17_I_0.83_)_3_ film	2.0 × 10^−6^	≈27	59.9	‐	[86]
Cs_0.05_FA_0.79_MA_0.16_Pb(I_0.8_Br_0.2_)_3_ film	≈2.0 × 10^−5^	0	≈3.7	12 000	[87]
Cs_3_Bi_2_I_9_ SC	‐	‐	1.65 ×10^3^	1.3×10^8^	[88]
Cs_2_AgBiBr_6_ SC	6.3 × 10^−3^	25	105(50)	59.7	[55]
FAMACs SCs	‐	‐	≈3.5 ×10^6^	42	[89]
Cs_0.15_FA_0.85_PbI_3_ and Cs_0.15_FA_0.85_Pb(I_0.15_Br_0.85_)_3_	‐	‐	‐	≈13.8	[90]
GAMAPbI_3_ SC	1.3 × 10^−2^	‐	23 000	16.9	[91]
(3AMPY)Pb_2_I_6_ SC	1.2 × 10^−4^	‐	207(50)	259100	[92]
MAPbX_3_ cascade SC	1.285 × 10^−3^	660	1580	‐	[93]
CsPbBr_3_ SC	‐	50	770(40)	‐	[94]
CsPbBr_3_ SC	2.5 × 10^−3^	20	1256(80)	‐	[95]
Cs_0.99963_Rb_0.00037_PbBr_3_ SC	7.2 × 10^−4^	8	8097(50)	‐	[96]
CsPbBr_3_ film	1.32 × 10^−2^	50	55 684	215	[97]
CsPbBr_3_ film	‐	455	11840	‐	[98]
CsPbBr_3_ film	1.32 × 10^−2^	20.1	55 684 (30)	‐	[97]
CsPbBr_3_ film	‐	12 000	1450(45)	500	[99]
CsPbBr_3_ film	‐	Self‐driven	470	53	[100]
CsPbBr_3_ QDs	‐	0	1450(0.8)	17200	[6]
CsPbI_3_ SC	3.63 × 10^−3^	41.7	2370	219	[22]
Cs_3_Bi_2_I_9_ SC	7.97 × 10^−4^	50	1652.3	130	[101]
Cs_3_Bi_2_I_9_ SC	2.03 × 10^−5^	50	111.9(80)	‐	[56]
Cs_2_AgBiBr_6_ SC	6.3 × 10^−3^	25	105(50)	59.7	[55]
Cs_2_AgBiBr_6_ SC	1.94 × 10^−3^	2.5	288.8(50)	‐	[102]
Cs_2_AgBiBr_6_ SC	5.95 × 10^−3^	0.5	1974(50)	45.7	[103]
Cs_2_AgBiBr_6_ Wafer	5.51 × 10^−3^	2.5	250(50)	95.3	[104]
Cs_2_AgBiBr_6_ SC	1.65 × 10^−5^	5	16(70)	‐	[105]
Cs_2_AgBiBr_6_ SC	‐	6	316	‐	[21]
(Cs_0.98_Rb_0.02_)_2_AgBiBr_6_ SC	3 × 10^−5^	10	32(70)	27,4900	[105]
(Cs_0.98_K_0.02_)_2_AgBiBr_6_ SC	2.25 × 10^−5^	10	8(70)	‐	[105]
(Cs_0.98_Na_0.02_)_2_AgBiBr_6_ SC	2.25 × 10^−5^	10	1(70)	‐	[105]
(NH_4_)_3_Bi_2_I_9_ SC	1.1 × 10^−2^	10	8200(50)	55	[106]
Cs_2_TeI_6_ Film	5.2 × 10^−5^	2.2	19.5(40)	‐	[107]
Cs_x_FA_1‐x_PbI_3_ SC		400	2772.1(50)	‐	[108]
MA_3_Bi_2_I_9_ SC	2.87 × 10^−3^	60	1947(40)	83	[57]
MA_3_Bi_2_I_9_ SC	2.8 × 10^−3^	60	10 620(100)	0.62	[109]
MA_3_Bi_2_I_9_ Crystallites	4.6 × 10^−5^	2100	563(45)	9.3	[110]
Rb_3_Bi_2_I_9_ SC	2.51 × 10^−3^	2000	159.7(50)	8.3	[111]
(F‐PEA)_2_PbI_4_ SC	5.1 × 10^−4^	1	3402(120)	23	[40]
(BA)_2_CsAgBiBr_7_ /Cs_2_AgBiBr_6_	‐	Self‐driven	206(40)	‐	[5]
(BA)_2_(MA)_2_Pb_3_I_10_ Film	‐	‐	10860	69	[112]
BA_2_EA_3_Pb_3_Br_10_ SC	1.0 × 10^−2^	0	6800(70)	5500	[113]
(DMEDA)BiI_5_ SC		4 940	72.5(50)	‐	[114]
BDAPbI_4_ SC	4.43 × 10^−4^	494	242(40)	430	[115]
(F‐PEA)_2_PbI_4_ SC	5.1 × 10^−4^	≈1333	3402	23	[40]
MA_3_Bi_2_I_9_ SC	‐	28 600	872	31	[116]
(NH_4_)_3_Bi_2_I_9_ SC(//)	1.1 × 10^−2^	22	8 200	210	[106]
(NH_4_)_3_Bi_2_I_9_ SC(⊥)	4.0 × 10^−3^	65	803	< 55	[106]
(BA)_2_Cs_3_AgBiBr_7_ SC	1.21 × 10^−3^	10	4.2(70)	‐	[117]
(DMA)MAPbI_3_ SC	‐	4.2	12 000(8)	16.9	[91]
(GA)MAPbI_3_ SC	‐	4.2	23 000(8)	16.9	[91]
DABCO‐NH_4_Br SC	‐	‐	173(29)	4960	[118]
(CPA)_4_AgBiBr_8_ SC	‐	10	0.8(70)	‐	[119]
Cs_2_AgBiBr_6_ film	‐	≈16 667	18 000	145.2	[120]
Cs_2_AgBiBr_6_/polyvinyl alcohol film	‐	40 000	40	‐	[121]
Polymer‐Encapsulated Cs_4_PbI_6_ Thin Film	‐	‐	256.20(30)	‐	[121]
Cs_2_TeI_6_ polycrystalline ‐polyimide substrate	‐	‐	76.27(20)	170	[122]
Cs_2_TeI_6_ flexible polyimide substrate	‐	6.67 × 10^3^	226.8(50)	73 090	[123]
*α*‐Se	5.0 × 10^−10^	10^4^	20(20)	5500	[124]
HgI_2_	5.0 × 10^−9^	250	1600(70)	10 000	[125]
CdZnTe polycrystalline	7.0 × 10^−9^	250	2400(80)	50 000	[126]
CdZnTe crystal	3 × 10^−3^	‐	‐	‐	[127]
CdTe SC	5.178 × 10^−3^	500	4.2 × 10^5^(61)	‐	[128]

### 3.2 Rich Synthesis Approaches

#### 3.2.1 Synthesis Approaches of HP SC

**Figure 5:**
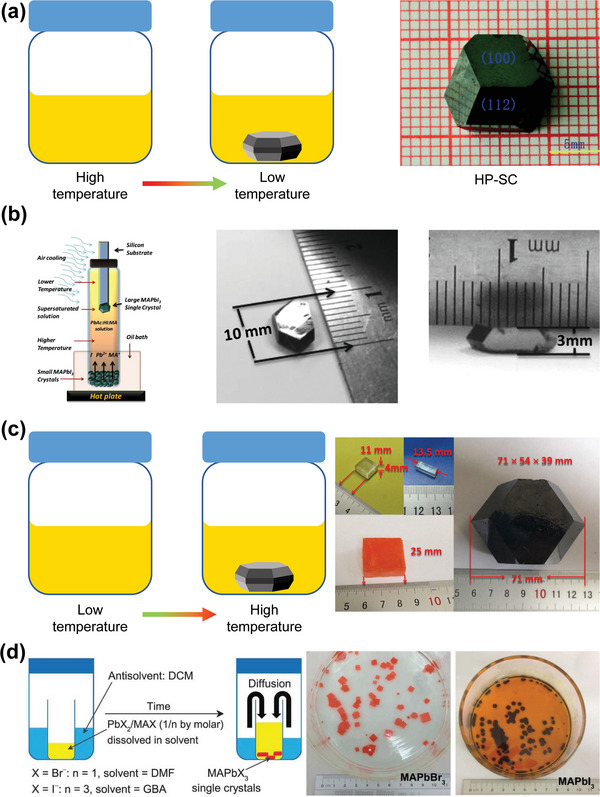
Methods used for HP SC growth and the basic mechanisms of these methods. a) STL method. Reproduced with permission.^[^ ^129^ ^]^ Copyright 2015, Royalty Society of Chemistry. b) TSSG method and the SC obtained by this method. Reproduced with permission.^[^ ^59^ ^]^ Copyright 2015, AAAS. c) ITC method. Reproduced with permission.^[^ ^130^ ^]^ Copyright 2015, John Wiley and Sons. d) AVC method. Reproduced with permission.^[^ ^132^ ^]^ Copyright 2015, AAAS.

The rich preparation methods of HP SC with high purity and large size, which are necessary to obtain a large *µτ* product. The methods of growing HP SCs can be divided into gas phase growth, solution growth, and solid growth. So far, the most popular growth method is solution growth. The solution growth methods can be divided into the solution‐temperature‐lowering (STL) method, the inversion crystallization (ITC) method, the antisolvent vapor‐phase‐assisted crystallization (AVC) method, and the solvent‐evaporation crystallization (SEC) method. In the STL method, the precursor solution prepared from HX aqueous solution (x = Cl, Br, I) will precipitate SCs when the temperature is reduced from ≈100 °C to room temperature, because the solubility of perovskite in HX‐based solvent will decrease with the decrease of solution temperature. For this method, bottom‐seed solution growth (BSSG) and top‐seed solution growth (TSSG) are typical methods widely used for cooling solutions. As shown in **Figure** **5**a, Dang et al. successfully obtained MAPbI_3_SCs (10 mm × 10 mm × 8 mm) at the bottom of the flask.^[^ ^129^ ^]^ Dong et al. obtained the same SCs (10 mm × 3.3 mm) with lateral thickness of 10 and 3.3 mm on a silicon substrate immersed in the top of the precursor solution (Figure 5b).^[^ ^59^ ^]^ In addition, HP SC also shown reverse dissolution behavior in some specific organic solvents. For example, in *γ*‐butyrolactone (GBL), dimethylformamide (DMF) and dimethyl sulfoxide (DMSO), SC has low solubility at high temperature. Utilizing this unusual solubility characteristic, Liu et al.^[^ ^130^ ^]^ and Saidaminov et al.^[^ ^131^ ^]^ grew MAPbI_3_, MAPbBr_3_, and MAPbCl_3_ perovskite SCs, as shown in Figure 5c. Their works also shown that DMF and GBL were suitable solvents for the growth of bromide and iodine SCs, and DMSO was the best solvent for the growth of chloride SCs. The AVC method is based on the differing solubility of perovskite in various solvents. In this method, two miscible solvents are usually used, where one is the main solvent for dissolving a large amount of raw materials, and the raw materials are insoluble in the other solvent (i.e., antisolvent). Therefore, the antisolvent can be added to the saturated precursor solution to rapidly reduce the solubility of the material and lead to crystallization. As shown in Figure 5d, Shi et al. first obtained millimeter‐sized MAPbBr_3_ and MAPbI_3_ SCs with this method by using dichloromethane (DCM) as the antisolvent.^[^ ^132^ ^]^ The SEC method is the simplest of the four widely used methods for perovskite SCs solution growth. In this method, a supersaturated solution can be achieved by evaporating the solvent at room temperature or heating the solution at a fixed temperature.

#### 3.2.2 Synthesis Approaches of HP Polycrystalline Thin Film

The cost of high‐efficiency devices manufactured by SC growth or polycrystalline thin‐film deposition processes is also low, because they are all completed by low‐cost solution processes.^[^ ^61^, ^62^ ^]^ At the initial stage of photovoltaic and optoelectronic HP research, polycrystalline perovskite thin films were coated using one‐step spin coating.^[^ ^133^, ^134^ ^]^ In 2014, an anti‐solvent drip strategy was developed for highly uniform HP thin films. In this method, anti‐solvents such as toluene and chlorobenzene are dropped onto the HP during the spinning of the precursor solution.^[^ ^135^, ^136^ ^]^ This simple method leads to a large area of uniform HP thin films.

**Figure 6:**
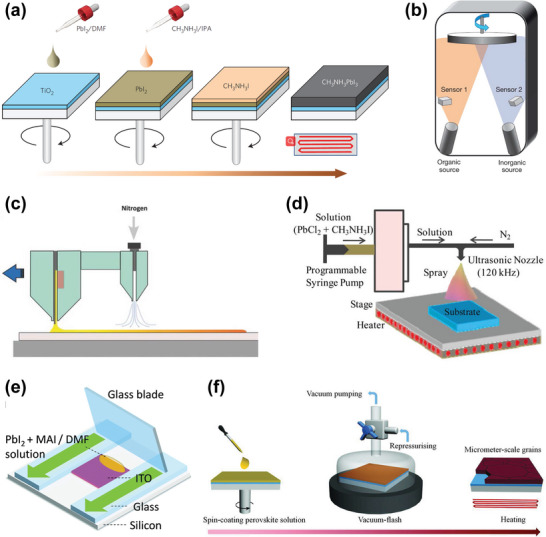
The deposition methods for forming diverse perovskite films. a) Sequential deposition by spin‐coating in two steps. Reproduced with permission.^[^ ^135^ ^]^ Copyright 2014, Springer Nature. b) Co‐evaporation deposition. Reproduced with permission.^[^ ^139^ ^]^ Copyright 2013, Springer Nature. c) Slot‐mold coating. Reproduced with permission.^[^ ^141^ ^]^ Copyright 2015, John Wiley and Sons. d) Spray‐coating deposition. Reproduced with permission.^[^ ^142^ ^]^ Copyright 2015, American Chemical Society. e) Doctor‐blade coating. Reproduced with permission.^[^ ^8^ ^]^ Copyright 2022, American Chemical Society. f) Vacuum flash‐assisted solution process. Reproduced with permission.^[^ ^143^ ^]^ Copyright 2016, AAAS.

Compared with the one‐step spin coating of perovskite precursor solution,^[^ ^137^, ^138^ ^]^ polycrystalline films can be prepared by a two‐step method, in which the inorganic and organic constituents are sequentially deposited (**Figure** **6**a),^[^ ^135^ ^]^ and the rapid and complete conversion of perovskite films with controllable crystal growth orientation and outstanding device performance can be realized. Uniform and flat HP polycrystalline thin films can also be obtained by vapor deposition. As shown in Figure 6b,^[^ ^139^ ^]^ a large‐area perovskite thin film was obtained by the co‐evaporation of inorganic and organic components and demonstrated excellent multilayer interface characteristics. The efficiency of solution‐treated perovskite solar cells exceeds 25%,^[^ ^140^ ^]^ which is equivalent to the high‐quality solar cells using single‐junction silicon currently dominating the photovoltaic realm. The demand for scalable and rapid processing of large‐area thin films is increasing. Therefore, slot‐mold coating, spray‐coating deposition, and perovskite doctor‐blade coating have been developed as alternatives to the unscalable spin‐coating strategy (Figure 6c–e).^[^ ^8^, ^141^, ^142^ ^]^ For example, the doctor‐blade coating process for large‐area deposition of HP films provides several attractive features including simplicity, low‐temperature and large‐scale deposition, and compatibility with roll‐to‐roll manufacturing. In addition, Li et al. designed a new method called the vacuum flash‐assisted solution process to prepare smooth perovskite polycrystalline films with high crystallinity, as shown in Figure 6f.^[^ ^143^ ^]^ Compared with the reported deposition methods employing anti‐solvent, vacuum‐flash treatment promotes the rapid crystallization of perovskite mesophase and solves limitations in the perovskite solution treatment process. In addition, the spatial heterogeneity and environmental toxicity were improved in the process of large‐area dissolution.^[^ ^135^, ^144^ ^]^

### 3.3 Designability of Materials Properties

In the semiconductor field, the intrinsic properties of active materials, including their crystal structure, band gap dispersion, defect density of states, emission characteristics, carrier lifetime, and mobility, are important for optoelectronic materials. HP materials usually exhibit excellent photo‐responsive properties such as high absorption coefficient, long carrier lifetime, and tunable band gap. These excellent properties along with the wide tunability of electronic structures make HPs highly designable for X‐ray detection.

#### 3.3.1 1. Adjustable Band Gap and Crystal Structure

Compared with the conventional oxide‐based perovskite (3.0–5.0 eV), the band gap of HPs lies in the visible light region (1.2–3.0 eV).^[^ ^145^, ^146^ ^]^ The main advantage of HPs is that is simple to adjust the electronic structure, which makes it easy to modify their band gap to suit the target application. At present, the most widely used calcite optical receptors including MAPbI_3_ (1.55 eV) and FAPbI_3_ (FA = CH(NH_2_)_2_) (1.43–1.48 eV) are close to 1.40 eV, which is the optimal band gap value according to the Shockley–Queisser efficiency limit. So far, the use of perovskite with a band gap of ≈1.45 eV as the active absorber has improved the efficiency of solar cells to more than 25%, which is the highest reported for a perovskite‐based solar cell.^[^ ^140^ ^]^

**Figure 7:**
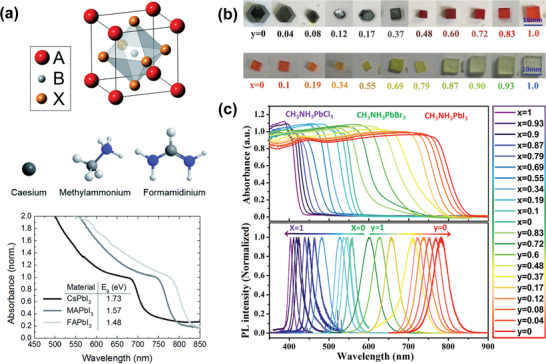
Absorbance behaviors of HP materials as the ions of ABX_3_ positioned at the A, B, and X sites are changed. a) The structure and absorption spectra of ABX_3_ in which A can be inorganic (e.g., Cs^+^) or organic cations (e.g., MA^+^ and FA^+^). Reproduced with permission.^[^ ^147^ ^]^ Copyright 2017, Royalty Society of Chemistry. b) Photographs of mixed‐halide HP SCs incorporating I, Br, and Cl. c) UV–Vis–NIR absorption spectra of mixed‐halide HP SCs. Reproduced with permission.^[^ ^148^ ^]^ Copyright 2017, Springer Nature.

In 2014, Eperon et al. studied the effect of substituting Cs^+^ or FA^+^ cations for MA^+^ cations at the A site (**Figure** **7**a).^[^ ^147^ ^]^ The organic cations at the A site overlap with the electron orbits of the BX_6_ octahedral phase, so there is almost no effect on the major electronic characteristics of the HP. However, within the range of suitable tolerance factor, the size effect of various cations will change the band gap of 3D perovskite due to the different degrees of lattice distortion. Also, the tolerance factor increases with larger A‐site cation radius, *r*_A_, which results in high stacking symmetry, leading to a decrease of the band gap and a red‐shift of the absorption edge. When a larger A cation of ABX_3_ is involved, the HP forms a low‐dimensional structure.^[^ ^2^ ^]^ The change of halide anion, X, including I^−^, Br^−^, Cl^−^ can also significantly change the band gap of HP. For MAPbX_3_, when the halogen atoms are varied from I to Br and Cl, the band gap has a wide controllable range from 1.55 to 3.11 eV. This indicates that the absorption spectrum and emission spectrum can be tuned over the whole visible range.^[^ ^130^ ^]^

Liu et al. studied the mixed‐type HP SCs incorporating I, Br, and Cl. (Figure 7b).^[^ ^148^ ^]^ For MAPbI_3‐_ *_x_* Br*_x_* crystals, the crystal color ranges from black to dark brown, red, and then orange. For MAPbBr_3‐_ *_x_* Cl*_x_* crystal, with the increase of *x*, the crystal color changes from orange to olive, yellow, and then becomes transparent. They demonstrated that mixed‐halide HPs can be successfully produced by replacing I with Br or by replacing Br with Cl. It can be inferred that as the interaction between lead ions and halide ions gradually mediates, the band gap of HPs can be finely tuned by changing the halide atom ratio. The optical results almost match the theoretical analysis. Figure 7c shows the absorbance spectra of bihalide perovskite samples. The results show that the initial absorption wavelength varies monotonically in the range of 428–843 nm with the change of halide composition.^[^ ^148^ ^]^

With the change of crystal structure and band gap, the optoelectronic properties including carrier accumulation and transport and resistivity of HP materials will also change greatly. For example, Liu et al. obtained crystal structures from 3D to 2D and then to 0D by adjusting the composition of HPs, and confirmed that the carrier mobility and resistivity changed significantly.^[^ ^57^ ^]^ This provides more opportunities for direct X‐ray detection to improve stability and optimize detection performance.

#### 3.3.2 High Photoluminescence Quantum Yield

**Figure 8:**
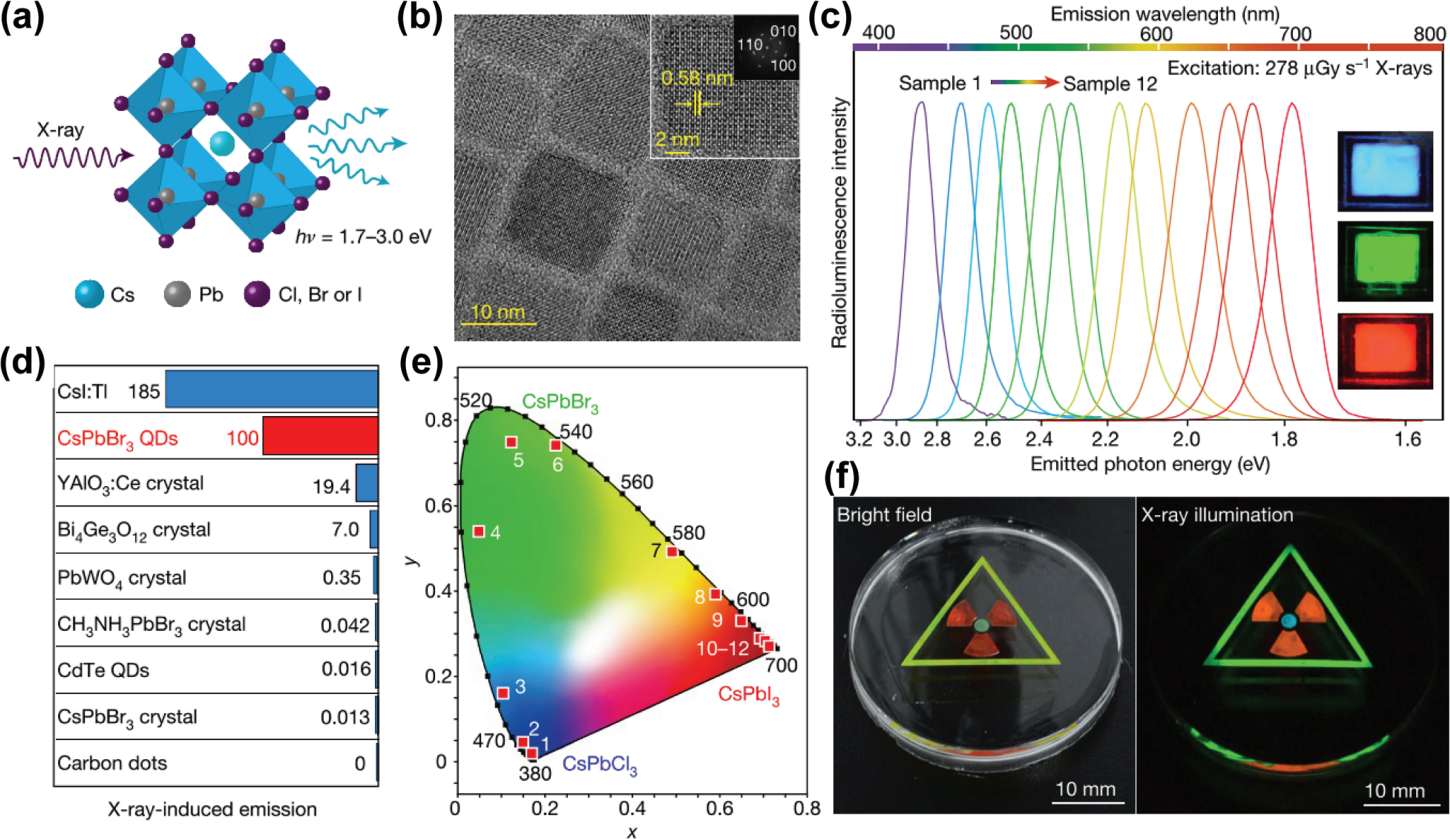
a) Schematic diagram of X‐ray‐induced luminescence in a lattice with a cubic all‐inorganic perovskite crystal structure. b) TEM micrograph of the prepared CsPbBr_3_ nanocrystals. c) Tunable X‐ray‐induced luminescence spectra of the perovskite quantum dots (QDs). d) Optical sensitivity of perovskite QDs scintillators versus non‐perovskite scintillator materials under X‐ray illumination. e) Commission Internationale de l'Eclairage (CIE) chromaticity coordinates of the X‐ray‐induced visible emissions measured for CsPbX_3_ nanocrystals. f) Multicolor scintillation of nanocrystal scintillators induced by X‐rays. Reproduced with permission.^[^ ^53^ ^]^ Copyright 2018, Springer Nature.

In the indirect detector, to avoid post‐scintillation energy loss, the emission band of the scintillator should agree with the optoelectronic spectral sensitivity of the combined photodetector. For the bulk of traditional scintillators, however, it is difficult to achieve multi‐color display under X‐ray irradiation because of their inherent non‐tunable emission characteristics. As discussed above, the band gap of HP can be conveniently modulated by mixing cations or halide ions, so that the luminescence spectra cover the entire visible range. Because the band gap is adjustable,^[^ ^149^ ^]^ Chen et al. was able to synthesize all‐inorganic HP CsPbX_3_ (X = Cl^−^, Br^−^, I^−^, or mixed halide) nanocrystals (**Figure** **8**a,b) that exhibited tunable narrow and colorful emission upon X‐ray excitation.^[^ ^53^ ^]^ Figure 8c shows the optical band gap of HPs in the range of 1.6–3.1 eV, which is far lower than those of traditional scintillators (such as CsI, 6.4 eV, NaI, 5.9 eV). Since the high photoluminescence quantum yield (PLQY) of scintillators decrease when the optical band gap energy increases, the light production of halogenated perovskite with low band gap can reach 129 000–250 000 photons MeV^−1^ (Figure 8d). It is worth noting that nearly uniform PLQY in the ultraviolet to near‐IR spectral range have been achieved in CsPbX_3_ nanocrystals (Figure 8e,f). This high PLQY is a promising characteristic for achieving high LY under X‐ray irradiation.^[^ ^150^ ^]^

#### 3.3.3 Lead‐Free Perovskite can be Achieved

**Figure 9:**
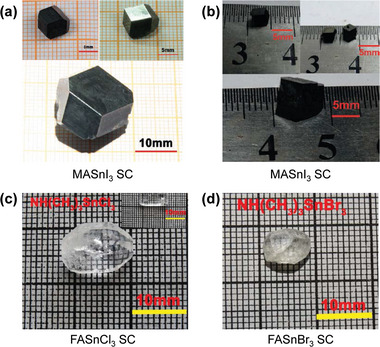
a) MASnI_3_ Reproduced with permission.^[^ ^154^ ^]^ Copyright 2016, John Wiley and Sons. b) FASnI_3_ SCs obtained by optimizing crystal growth conditions. c) FASnCl_3_ and d) FASnBr_3_ SC. Reproduced with permission.^[^ ^155^ ^]^ Copyright 2016, American Chemical Society.

The long‐term stability of lead‐based HPs and the impact of heavy metal content on the environment prompt the design of green perovskite materials. In recent years, the research progress and scientific problems of lead‐free perovskite have received extensive attention. These works emphasize the necessity of developing new perovskite materials with direct band gaps. One lead‐free perovskite candidate material appearing frequently is MASnI_3_.^[^ ^151^, ^152^ ^]^ MASnX_3_ (X = I, Br, Cl) perovskite thin films were prepared by a steam‐assisted solution method and heat‐injection method. Interestingly, excessive SnX_2_ will generate a secondary phase, which in turn will help improve the air stability of the device.^[^ ^153^ ^]^ In addition, Dang et al. reported the bulk crystal growth and different crystal morphology of orthogonal hybrid perovskite MASnI_3_ and FASnX_3_ (X = Cl, Br) by the solution growth method in an ambient atmosphere, as shown in **Figure** **9**. In addition, they reported detailed structure determination and refinement, phase transition, band gap, band structure calculation, nonlinear optical properties, and stability of these SCs.^[^ ^154^, ^155^ ^]^

### 3.4 Low Cost

The raw materials utilized for forming HPs are rich in nature and thus are low cost. According to suppliers such as Aladdin and Sigma‐Aldrich, the entire material cost for perovskite SC is in the range of $2.65–$3.65 per cm^3^, which is much lower than those of commercial scintillators such as YAG:Ce (25 $ g^−1^), LYSO (8.4 $ g^−1^), and YAP:Ce (60 $ g^−1^). The low cost of HP scintillators is also comparable to that of CsI:Tl (2.5 $ g^−1^). Once production of HPs scales up, their material cost may decrease by 5–10 times. Thus, the projected cost of HP SCs material is calculated as <$0.3 per cm^3^, which is much cheaper than commercial CZT crystals ($0.9–$1.2 per cm^3^).^[^ ^15^ ^]^

## 4 Advancements in HP Direct‐Type X‐Ray Detectors

### 4.1 Device Structures and Electrodes for Direct X‐Ray Detectors

After discussing the main parameters and mechanisms of direct‐type and indirect‐type conversion detection and the unique optoelectronic advantages of HPs in X‐ray detectors, herein, we introduce and discuss the latest progress of HP active layers for direct X‐ray detection in detail.

An ideal direct‐type device for X‐ray detection and imaging usually has the following three characteristics:^[^ ^10^ ^]^ i) sufficient sensitivity to X‐rays to form a high‐quality image at a given dose rate. ii) Low detection limit to allow reducing the exposure to X‐rays. iii) Ability to remain stable for a long time under high‐energy irradiation. In order to achieve decent device performance, the active semiconductor materials in the direct‐type detector involve the following features, aside from involving heavy elements (Pb, Bi, et al.), to interact effectively with X‐rays:^[^ ^10^ ^]^ i) effective charge collection requires a large *µτ* product; ii) the trap density of the active layer must be minimized to decrease the dark current of the semiconductor; iii) for high‐bias operation, a large resistivity is necessary to maintain detection stability. This paper discusses the research progress of HP SC and polycrystalline thin‐film X‐ray detectors with different structures, including coplanar, and vertical.

**Figure 10:**
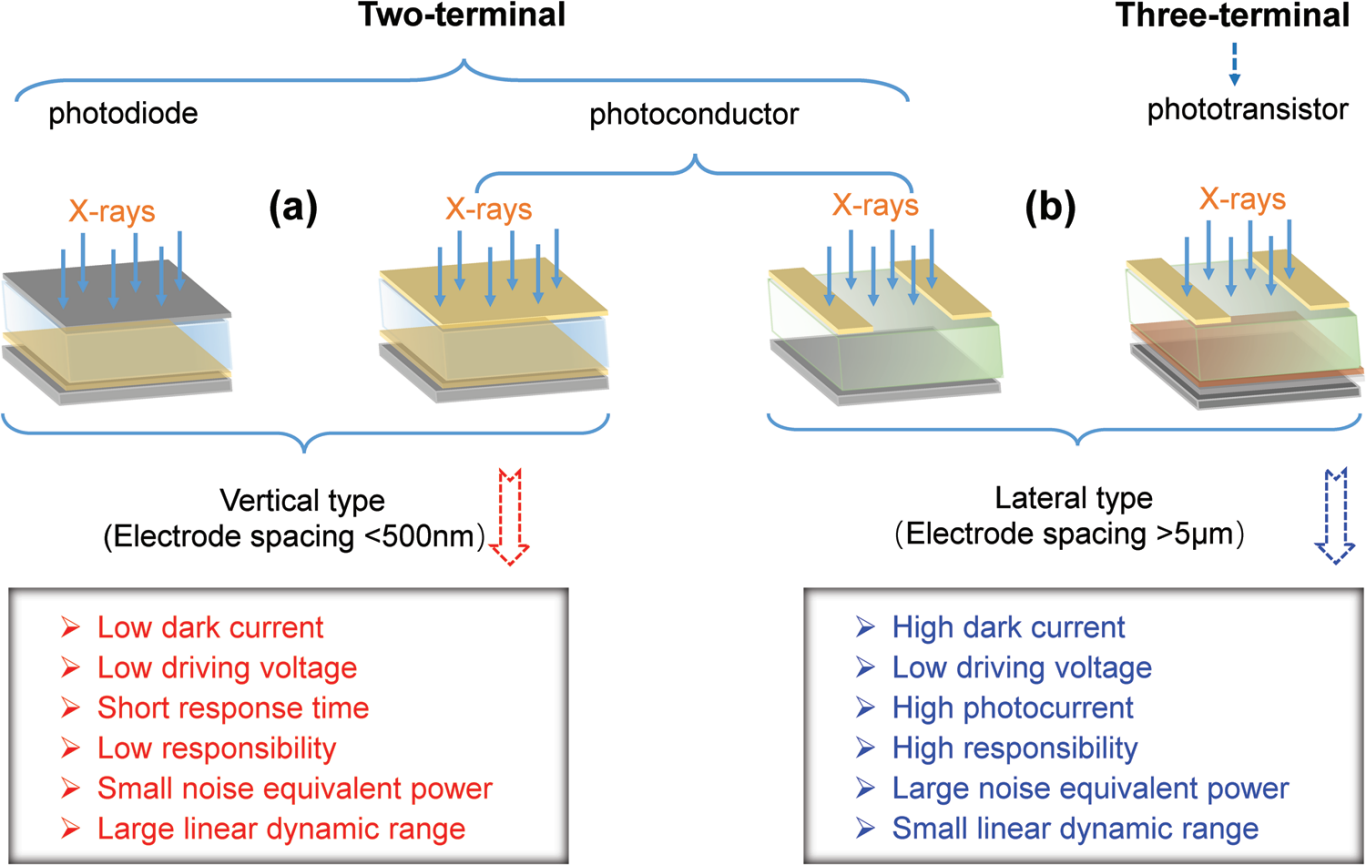
Schematic diagrams and the features of vertical and lateral architectures for direct‐type detection. a) Vertical devices with photodiode and photoconductor architectures; b) lateral devices with photoconductor and phototransistor architectures. Reproduced with permission.^[^ ^158^ ^]^ Copyright 2021, John Wiley and Sons.

According to different working mechanisms, perovskite photodetector devices are grouped into three basic structures: photodiodes, photoconductors, and phototransistors (**Figure** **10**).^[^ ^156^ ^]^ The architectures of photodiodes and photoconductors are 2‐terminal, in which the active absorber material is sandwiched between two conductive metal electrodes (e.g., Au, Ag). Photodiodes usually contain Schottky, p‐n or p‐i‐n junctions. Their architecture is similar to the photovoltaic devices. Generally, perovskite photodiodes are widely used in vertical device structures, as presented in Figure 10a. The junction barrier in a photodiode can easily result in low noise current and dark current as well as fast detection responsivity, and the electrons and holes in a photodiode can be separated even under a lower driving voltage. Furthermore, the photodiode shows fast response and a large linear dynamic range. However, photodiodes have lower spectral responsivity and external quantum efficiency.^[^ ^157^ ^]^ In contrast, photoconductor devices usually contain an optically active perovskite absorbing layer and two ohmic metal electrodes (Figure 10b), known as the simplest architecture. In the perovskite absorber, carriers generated by light are separated by the driving voltage, then collected and transported by two metal electrodes. So a photoconductor requires an applied driving voltage. The geometric configuration is either vertical or coplanar. In the vertical‐type devices, the top and bottom electrodes sandwich the active absorption layer. This structure is applied widely because of its convenient integration with a substrate on a pixel matrix. For the coplanar‐type devices, the radiation directly impinges upon the active layer and the electrodes located laterally at interfaces. The lateral HP photoconductor device structure (Figure 10b) has received more attention because of its simpler process of manufacture. In addition, the lateral device structure does not require a transparent electrode.^[^ ^158^ ^]^ Because the electric field strength in the photoconductor is only related to the electrode spacing, not the layer thickness, it requires a lower driving voltage. Meanwhile, the photoconductor of the lateral device structure has a large electrode spacing, which renders a slow response rate and high applied voltage. In comparison with the lateral‐type architecture, the electrode spacing of the vertical structure is small, which is conducive to reducing the driving voltage and response time of the photoconductor. Phototransistors generally are three‐terminal devices, like field‐effect transistors, where the drain and optical source, dielectric layer and semiconductor channel are involved in a phototransistor device, as presented in Figure 10b. Different from a photoconductor, changing the gate voltage of a phototransistor can modulate the conductance of the semiconductor channel under light or dark conditions, which is conducive to amplifying the photocurrent or reducing the dark current, so as to improve performance parameters such as spectral responsivity, EQE and specific detection rate.^[^ ^159^ ^]^ As described above, the X‐ray conversion process in the three detector architectures will be different due to different operating parameters and material properties.

**Figure 11:**
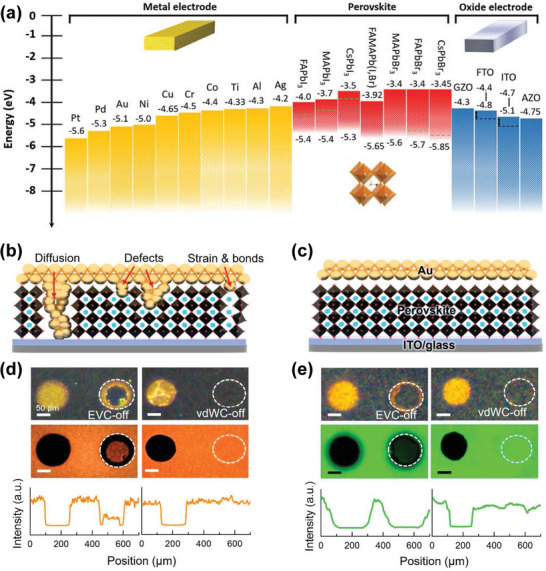
Perovskite and metal electrode contact interface analysis. a) Energy levels of the well‐studied HPs, metals, and oxide electrodes. Reproduced with permission.^[^ ^160^ ^]^ Copyright 2022, John Wiley and Sons. b,c) Cross‐section schematics of metal electrodes on perovskite thin films made by thermal evaporated and van der Waals contact methods. d,e) The optical images, PL mapping, and line scan profiles of the PL mapping intensity for the samples in (c,d). Reproduced with permission.^[^ ^161^ ^]^ Copyright 2022, American Chemical Society.

As discussed above, HP X‐ray detectors can be fabricated into a variety of different structures. In most cases, the electrodes, especially metal electrodes, are contacting with perovskite directly. Due to the ionic nature and highly reactive interface of HPs, the contact characteristics between electrodes and perovskites are significantly for achieving desirable photoelectronic performance in devices based on perovskite materials. Therefore, in addition to improving the quality of HPs, optimizing the properties of the interface between perovskite and electrode is equally important. Similar to other semiconductors, the energy arrangement in device plays a crucial role in determining the performance. In HP X‐ray detectors, it seems that the energy levels of various electrodes meet the requirements of device energy level matching. **Figure** **11**a shows the energy levels of HPs, the common metal, and conductive metal oxide electrodes. However, in the device preparation, because the nature of the ionicity of HP makes it exist interfacial electrochemical reaction with the electrode, Fermi level pinning and ion accumulation, which is unfavorable to the device performance.^[^ ^160^ ^]^

Besides, since the metal electrodes of HP X‐ray detectors are usually prepared by the thermal evaporation physical vapor deposition (PVD) process. In the process of electrode preparation, high‐energy metal atoms generated by PVD will directly bombard the perovskite surface, thus causing non‐negligible damage to the device (Figure 11b). The defects introduced by the damage of the interface between electrode and HP will lead to serious recombination of photogenerated carriers and then affect the stability of the device. This problem was confirmed by Lee et al. by using the space charge‐limiting current method.^[^ ^161^ ^]^ It is found that the average defect density of devices with metal electrodes fabricated by the PVD process increased by 26–48%. In addition, interface defects lead to easy migration of ions under electric field, which seriously affects the performance and stability of the photodetector. In order to solve this problem, they adopted the physical lamination van der Waals metal preparation method, which effectively reduced the interface defects (Figure 11c). In situ photoluminescence test shows that the defects in the devices are significantly reduced when the metal electrodes are fabricated by physical lamination (Figure 11d,e). Therefore, in the preparation of HP X‐ray detector, non‐destructive electrode preparation techniques such as physical lamination and electrode transfer can be adopted to further improve the performance and stability of the detector.

### 4.2 HP Polycrystalline Thin Film for Direct X‐Ray Detectors

#### 4.2.1 Vertical Structure

**Figure 12:**
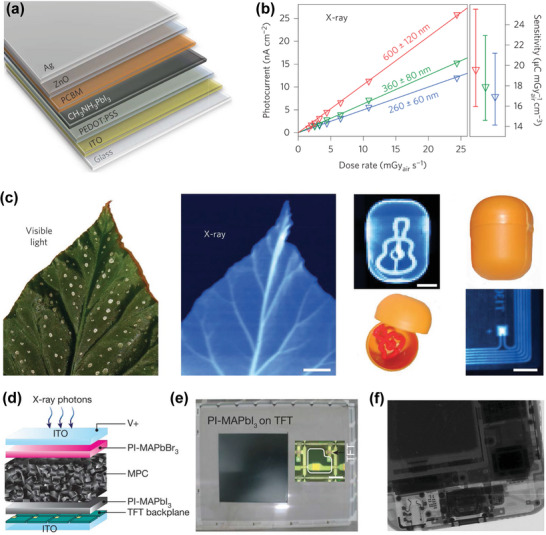
a) Schematic diagram of MAPbI_3_ detector with vertical structure. b) Photocurrent density as a function of X‐ray dose rate. c) Photograph (left) of a leaf and the corresponding X‐ray image (center) formed by the photoconductor along with photographs and X‐ray images of other samples. Reproduced with permission.^[^ ^79^ ^]^ Copyright 2015, Springer Nature. d) Illustration of a vertical structure for HP X‐ray detector. Reproduced with permission.^[^ ^35^ ^]^ Copyright 2015, Springer Nature. e) The X‐ray image of PI‐MAPbI_3_ on a TFT backplane (*α*‐Si:H) by spin casting. f) X‐ray image of part of a smartphone observed in the digital detector. Reproduced with permission.^[^ ^35^ ^]^ Copyright 2017, Springer Nature.

Yakunin et al. prepared a direct‐type X‐ray detector with photovoltaic architecture (vertical structure) utilizing HP polycrystalline thin films for the first time.^[^ ^79^ ^]^ In the photovoltaic devices, MAPbI_3_ film with different thicknesses (<1 µm) formed by spin coating was exploited as the active layer. X‐ray sensitivity of 25 µC mGy^−1^ cm^−3^ was achieved, which is higher than that of *α*‐Se (17 µC mGy^−1^ cm^−3^) (**Figure** **12**a,b). The photoconductive detector, with a 60 µm‐thick MAPbI_3_ film active layer, has fast response to the X‐ray. Compared with the photoelectric device using the sub‐micron MAPbI_3_ active layer, the sensitivity of the photoconductive device (7 µA cm^−2^) increased by more than 100 times on X‐ray irradiating. Using this single device with photoconductive architecture, imaging with strong contrast and large dynamic range was achieved, as presented in Figure 12c.

Kim et al. demonstrated a high‐efficiency, large‐area, 830‐µm‐thick MAPbI_3_ X‐ray detector prepared by solution growth technology.^[^ ^35^ ^]^ An innovative approach of preparing perovskite polycrystalline thick films (20–100 µm) by an all‐solution method was also proposed, which can be used for blade‐coating printing on a large‐area TFT substrate. To decrease the dark current, two polyimide (PI) calcite composite layers were inserted via spin casting on the top and bottom of the absorption layer (Figure 12d,e). A large *µτ* product (1.0 × 10^−4^ cm^2^ V^−1^), high sensitivity (up to 1.1 × 10^4^ µC Gy^−1^ cm^−2^), and fast response to a short X‐ray pulse sequence (pulse width 50 ms) were obtained. This indicated that the photocurrent density is low. Taking advantage of these superior performances, X‐ray imaging of a smaller smartphone at a low dose of 10 µGy_air_ was successfully produced, as shown in Figure 12f. Due to effective solution‐based alloying, triple‐cation Cs perovskite shows special sensitivity. In recent years, people have explored a new type of Cs‐based triple‐cation hybrid high‐voltage thin‐film device, which has high X‐ray sensitivity and can be used for direct X‐ray detection. Under a reverse bias voltage (0.4 V), the sensitivity for X‐ray detection was further improved (97 ± 1 µC Gy^−1^ cm^−2^).^[^ ^157^ ^]^

#### 4.2.2 Co‐Planar Structure

**Figure 13:**
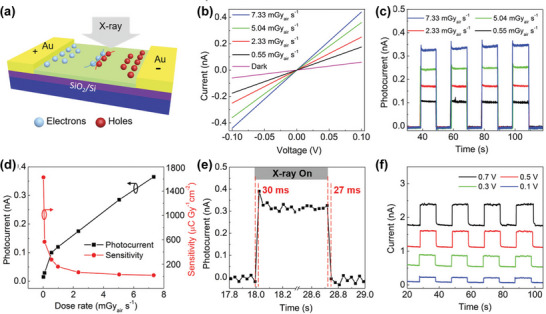
a) The key device fabrication procedure via an inkjet printing method. b,c) Device response curves under X‐ray irradiation with various X‐ray dose rates or none at a given bias voltage of 0.1 V. d) X‐ray sensitivity and photocurrent curves at different dose rates at a given bias voltage of 0.1 V. e) Temporal response curves of a device with dose rates of 7.33 mGy_air_ s^−1^ at a given bias voltage of 0.1 V. f) Device response curves at different bias voltages for X‐ray pulses with a dose rate of 7.33 mGy_air_ s^−1^. Reproduced with permission.^[^ ^6^ ^]^ Copyright 2019, John Wiley and Sons.

The co‐planar phototransistor and photoconductor generally sacrifice response speed in order to maintain high photocurrent because of the larger spacing between the electrodes, which leads to sluggish response speed even under high driving voltage. Despite this advantage, very little work has been reported to fabricate co‐planar X‐ray detectors with perovskite polycrystalline films, as they have relatively low carrier mobility. In addition, high quality thick polycrystalline films are difficult to prepare. Liu et al. Synthesized all‐inorganic HP CsPbBr_3_ quantum dot thin film by the solution method and prepared a large‐area X‐ray detector array on SiO_2_/Si substrate by inkjet printing (**Figure** **13**a). The dark current and soft‐X‐ray‐induced photocurrent was detected in the lateral configuration, as shown in Figure 13b,c. At the irradiation dose rate of 0.0172 mGy_air_ s^−1^, the sensitivity of up to 1450 µC Gyair^−1^cm^−2^ was achieved at only 0.1 V bias voltage, which is higher than that of traditional *α*‐Se devices by a factor of ≈70 (Figure 13d–f).^[^ ^6^ ^]^

### 4.3 HP Single Crystal for Direct‐Type X‐Ray Detectors

A high density of traps in polycrystalline films including impurities, point defects, grain boundaries, and dislocations can result in non‐radiative recombination and material degradation. In contrast, the single crystal (SC) HPs have extremely low capture density (10^9^–10^10^ cm^−3^) and greatly enhanced stability.^[^ ^162^, ^163^ ^]^ Meanwhile, the activation energy of ion migration in SC HPs is markedly higher than that in polycrystalline material under a given dark current. This unique feature makes HP SCs attractive and promising in the high‐energy irradiation detection field. In recent years, perovskite SCs have rapidly advanced and surpassed traditional semiconductor optical materials in the growing field of X‐ray detection. Once the problem of the fabrication of high‐quality SCs by the controlled solution growth method has been solved, perovskite SCs will reach their full potential as excellent X‐ray detectors.

#### 4.3.1 Vertical Structure

##### 3D HP SC X‐Ray Detectors

**Figure 14:**
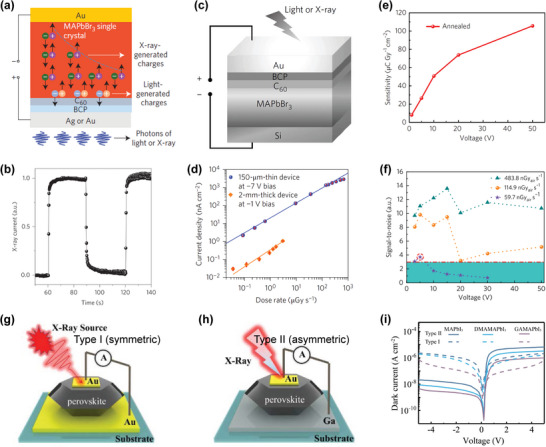
a) Device structure of the MAPbBr_3_ SC detector. b) Dynamic response curve to X‐rays in a MAPbBr_3_ SC detector. Reproduced with permission.^[^ ^71^ ^]^ Copyright 2016, Springer Nature. c) Device structure of the Si‐integrated MAPbBr_3_ SC detector. d) Dose rate dependent X‐ray‐generated photocurrent of the Si‐integrated MAPbBr_3_ SC detector. Reproduced with permission.^[^ ^70^ ^]^ Copyright 2017, Springer Nature. e) SNR of the Cs_2_AgBiBr_6_ SC X‐ray detector at different applied voltage. f) Sensitivity of the Cs_2_AgBiBr_6_ SC X‐ray detector. Reproduced with permission.^[^ ^55^ ^]^ Copyright 2017, Springer Nature. g,h) Device structures of the MAPbI_3_ based X‐ray detectors with the type I symmetric electrode and type II asymmetric electrode. i) Dark *I–V* curves of the detector with symmetric and asymmetric structure. Reproduced with permission.^[^ ^91^ ^]^ Copyright 2019, John Wiley and Sons.

The first direct X‐ray detector using perovskite SC was reported by Stoumpos et al. in 2013.^[^ ^28^ ^]^ The vertical Bridgman method was employed to grow CsPbBr_3_ SCs in a three‐zone furnace. The measured high electron µ*τ* product was 1.7 × 10^−3^ cm^2^ V^−1^, which is comparable with optoelectronically active cadmium telluride, and it also has a high value for holes, that is, 1.3 × 10^−3^ cm^2^ V^−1^, which is ≈10 times that of cadmium telluride. Since then, perovskite SCs as detector materials have undergone rapid progress. Wei et al. demonstrated the actual real‐time response of perovskite SC detectors to X‐rays for a 150‐µm‐thick photodiode structure of MAPbBr_3_ SC.^[^ ^71^ ^]^ **Figure** **14**a shows the dynamic response of this SC detector to X‐ray irradiation from an Ag tube at 50 kVp (Figure 14b). Moreover, the *µτ* reached 1.2 × 10^−2^ cm^2^ V^−1^, and the sensitivity was 80 µC Gy_air_^−1^cm^−2^.^[^ ^70^ ^]^

In the following few years, different kinds of lead and lead‐free perovskite SCs including all‐inorganic and organic and hybrid components were proved to be sensitive to high‐energy X‐rays. So far, the best detection performance of perovskite SC devices was reported by Wei et al.^[^ ^76^ ^]^ The sensitivity of their doped compensation MAPbBr_2.94_Cl_0.06_ SC detector was as high as 8.4 × 10^4^ µC Gy^−1^cm^−2^ with a low limit of detection (LoD) of 7.6 nGy s^−1^ (Figure 14c,d), suitable for detection of soft X‐rays (8 keV). Furthermore, the compensation provided by Cl^−^ dopants in p‐type MAPbBr_3_ SC was proved to significantly improve the volume resistivity and electron hole transport, which favors improvement of the CCE and maintaining low current under dark field conditions. Previous studies of lead‐free Cs_2_AgBiBr_6_ showed the sensitivity of the SC‐based detector decreased by an order of magnitude at 50 V, whereas the LoD value was as low as 59.7 nGy s^−1^ at 5 V (Figure 14e,f).^[^ ^55^ ^]^ Most perovskite irradiation detector devices involve millimeter‐size SCs, where a single pixel structure includes a perovskite SC sandwiched between two metal electrodes. There are two typical detector configurations, including type I and type II, as presented in Figure 14g,h. Symmetric electrodes are used in type I detectors, and small Schottky barriers for holes are formed at the junctions between the semiconductor and metals, which promotes injection of the holes into the MAPbI_3_ SC, resulting in large leakage current (Figure 14i).^[^ ^91^ ^]^ For the asymmetric architecture of the type II detector, a high Schottky barrier occurs at the interface of the MAPbI_3_ SC and Ga under reverse bias, which greatly suppresses carrier injection and significantly inhibits the leakage current.

**Figure 15:**
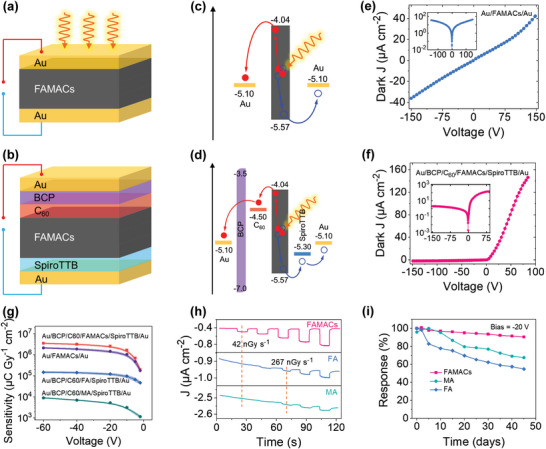
Schematic illumination of two types of FAMACs SC X‐ray detectors with different electrode configurations: a) symmetric type with Au electrodes at both sides and b) asymmetric type with SpiroTTB/Au and C60/BCP/Au serving as hole and electron collection electrodes. c,d) Energy levels and corresponding band diagrams of the two types of devices. e,f) *J–V* curves of the two kinds of X‐ray detectors under dark conditions. g) The relationship between the X‐ray sensitivity of the SC X‐ray detectors and the bias voltage. h) The current response of the SC X‐ray detectors. i) The long‐term stability of the SC detectors. Reproduced with permission.^[^ ^89^ ^]^ Copyright 2021, John Wiley and Sons.

To improve the long‐term stability of the 3D HP SC‐based X‐ray detectors, Liu's group further designed and manufactured FAMACs SC X‐ray detectors, as shown in **Figure** **15**a,b. Two kinds of electrode structures or device structures have been utilized, namely, symmetric electrodes with the device structure of Au/FAMACs SC/Au and an asymmetric one with the device structure of Au/BCP/C_60_/FAMACs SC/ spiroTTB/Au. The corresponding energy band diagrams of these two types of devices are shown in Figure 15c,d. It should be noted that the carrier injection has been suppressed in the asymmetric electrode device due to the energy barrier to holes or electrons at the anode and cathode. As a result, the FAMACs SC X‐ray detector with the asymmetric electrode structure delivered a dark current density as low as 1.44 µA cm^−2^ under a large reverse bias of −75 V (Figure 15e). The dark current density is ≈100× larger under 75 V bias voltage for the symmetric device, reaching a value as large as 133.33 µAcm^−2^. This is due to the rectified *J–V* curve of the p‐n/p‐i‐n diode device. Correspondingly, the dark current density of the asymmetric device is still as small as 2.03 µA cm^−2^ under −150 V bias. In contrast, at a bias of −150 V, the dark current density of the symmetric electrode‐based detector was much larger, reaching −6.06 µA cm^−2^ (Figure 15f). The statistical results also show that under the same bias of −150 V, the symmetric‐electrode detector has much higher dark current density, which is around 15× higher than that of the asymmetric electrode detector.^[^ ^89^ ^]^ These results show that choosing electrode materials with different Fermi levels can allow construction of effective junction‐type devices without the assistant of any further processing, such as thermal annealing or surface passivation. Therefore, under the illumination of the same X‐ray source, the asymmetric FAMACs SCs X‐ray detector obtained the highest sensitivity (3.5 ± 0.2) ×10^6^ µC Gy_air_^−1^ cm^−2^ (Figure 15g), which is ≈29 times the previous record of 1.22 × 10^5^ µC Gy_air_^−1^ cm^−2^. In addition, the FAMACs SC detectors delivered a low X‐ray detection limit of 42 nGy s^−1^ (Figure 15h) as well as stable current curves under both dark conditions and X‐ray illumination (Figure 15i). Since then, multiple A‐site ion mixed halide HP SCs have been successively reported.^[^ ^164^ ^]^ For example, Gao et al., by including more A‐site ions to reduce the defects and alloying B‐site metal ions to decrease the microstrain caused by A‐site substituting. Therefore, the X‐ray detectors made on the formed HP SCs show high sensitivity of (2.6 ± 0.1) × 10^4^ µC Gy_air_^−1^ cm^−2^, ultralow limit of detection of 7.09 nGy_air_ s^−1^, as well as excellent stability.^[^ ^165^ ^]^

##### 2D HP SC X‐Ray Detectors

**Figure 16:**
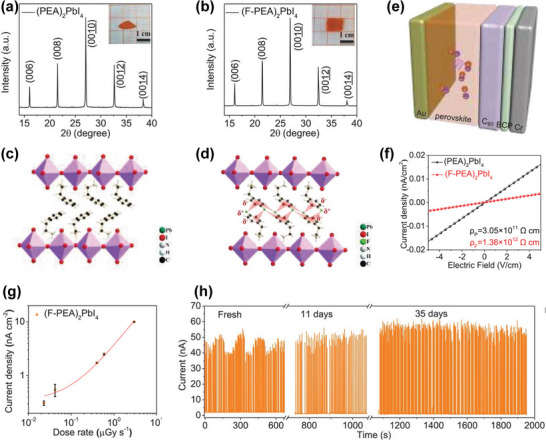
2D Perovskite SC X‐ray detector with vertical structure. a,b) Pictures and corresponding XRD of the 2D (PEA)_2_PbI_4_ SC and (F‐PEA)_2_PbI_4_ SC, respectively. c,d) The crystal structure of 2D (PEA)_2_PbI_4_ and (F‐PEA)_2_PbI_4_, respectively. e) Device structure of the 2D (PEA)_2_PbI_4_ SC and (F‐PEA)_2_PbI_4_ SC detector. f) Bulk resistivity determination of the 2D (PEA)_2_PbI_4_ SC and (F‐PEA)_2_PbI_4_ SC detector. g) Detection limit of the 2D (F‐PEA)_2_PbI_4_ SC X‐ray detector. h) Stability of the 2D (F‐PEA)_2_PbI_4_ SC X‐ray detector. Reproduced with permission.^[^ ^40^ ^]^ Copyright 2020, John Wiley and Sons.

Based on the adiscussion in last section, it can be seen that although 3D HP SC X‐ray detectors have high sensitivity, however, their internal ion migration induce serious impact on the stability and detection limit of the detector. Besides, in order to obtain enough CCE, large bias should be used on the detector, but the polarization effect is also increased in the X‐ray detector, that is, the detector shows high dark current and dark current drift under the condition of large bias. All these problems should be attributed to the notorious ions migration effect in perovskite devices.^[^ ^64^ ^]^ In such case, 2D perovskite seems to be the candidate that can be used to solve this problem, because their lead halide octahedral sheets are separated by large organic spacer, and demonstrated with suppressed ions migration. Even though the large organic cations can increase the activation energy of ions transport. However, their large dark current and long‐term operation stability problems are still challenges which limiting the X‐ray detection performance. Therefore, Wei et al., introduced deficient F atoms to enhance the supramolecular interactions as supramolecular anchor in the organic spacer (**Figure** **16**a–d), thus the ion migration path can be efficiently blocked and then the 2D SC further stabilized.^[^ ^40^ ^]^ In addition, the F atoms can also decrease the charge contraction. Therefore, the obtained (F‐PEA)_2_PbI_4_ SC shows a very high bulk resistivity of 1.36 × 10^12^ Ω cm and then realized a low noise in the SC detector (Figure 16e,f). All the above advantages enable the sensitive response to 120 keVp hard X‐rays from DR system with lowest detectable the (F‐PEA)_2_PbI_4_ SC shows high sensitivity of 3042 µC Gy_air_^−1^ cm^−2^, low detection limit of 23 nGy s^−1^ (Figure 16g) and excellent long‐term stability (Figure 16h) to 120 keVp hard X‐rays.

##### 1D HP SC X‐Ray Detectors

**Figure 17:**
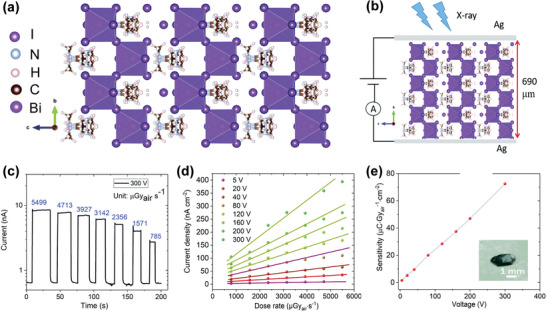
1D Perovskite SC X‐ray detector with vertical structure. a) Crystal structures of the 1D (DMEDA)BiI_5_. b) Device structure of the (DMEDA)BiI_5_ SC detector. c) X‐ray response current of the (DMEDA)BiI_5_ SC detector under different dose rate and bised at 300 V voltage. d) Dose rate dependent response current density of the (DMEDA)BiI_5_ SC detector for various bias voltages. e) Sensitivity of the (DMEDA)BiI_5_ SC detector at different biases. Reproduced with permission.^[^ ^114^ ^]^ Copyright 2020, Royalty Society of Chemistry.

Recently, the 3D bismuth halide perovskites such Cs_2_AgBiBr_6_ SC^[^ ^55^ ^]^ and 2D bismuth halide perovskites such (NH_4_)_3_Bi_2_I_9_ SC^[^ ^106^ ^]^ have been successively reported and demonstrated their low dark current, high X‐ray detection sensitivity as well as robust stability. Therefore, it is interesting to see how the X‐ray detection performance will be if the structural dimension is further reduced to 1D. Tang et al., further lowing the structure dimensionality to 1D and obtain the 1D CH_3_NH_3_CH_2_CH_2_NH_3_CH_3_BiI_5_ ((DMEDA)BiI_5_) SC using an aqueous HI method. Based on this 1D SC, they fabricated a photoconductive X‐ray detector with a vertical structure of Au/ (DMEDA)BiI_5_/Au (**Figure** **17**a).^[^ ^114^ ^]^ Because of the negligible ionic migration rate in the (DMEDA)BiI_5_ SC, the fabricated X‐ray detector shows a stable baseline (Figure 17b,c). Furthermore, a sensitivity of 72.5 µC Gy_air_^−1^ cm^−2^ was realized on the 1D HP SC (Figure 17d,e).

##### 0D HP SC X‐Ray Detectors

**Figure 18:**
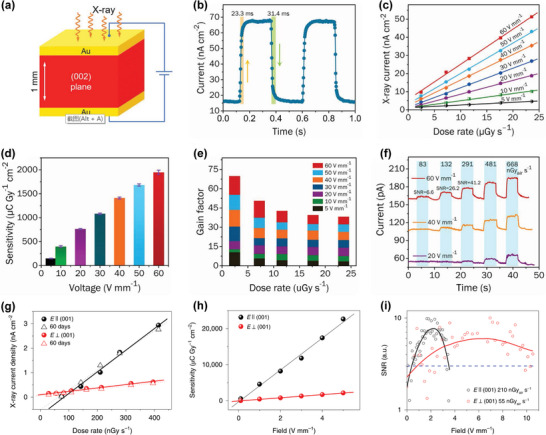
X‐ray detectors with lead‐free HP SC. a) Schematic diagram of a vertical‐type detector with 0D MA_3_Bi_2_I_9_ SC. b) Dynamic response to X‐rays in SC MA_3_Bi_2_I_9_. c) The relation curves of X‐ray response current with irradiation dose rates at different voltages (the slope of each fitting line represents the sensitivity). d) The optical sensitivity of SC MA_3_Bi_2_I_9_ under various given applied electric fields. e) Gain factor of a MA_3_Bi_2_I_9_ SC detector for various X‐ray dose rates under different electric fields. f) *I–t* curves of photocurrent response of the MA_3_Bi_2_I_9_ under various X‐ray dose rates and electric fields. Calculated signal‐to‐noise ratios are given. Reproduced with permission.^[^ ^57^ ^]^ Copyright 2020, Elsevier. g) Comparison of photocurrent densities of anisotropic (NH_4_)_3_Bi_2_I_9_ SC at different X‐ray dose rates and electric field orientations. Solid lines represent pristine conditions and dotted lines are after 60 days ageing under ambient conditions. h) Sensitivities of (NH_4_)_3_Bi_2_I_9_ SC to X‐rays. i) Signal‐to‐noise ratio of (NH_4_)_3_Bi_2_I_9_ devices with the electric field parallel and perpendicular to the 001 facet. Reproduced with permission.^[^ ^106^ ^]^ Copyright 2019, Springer Nature.

Ion migration in HP accelerates its structural decomposition, which leads to deterioration of device optoelectronic performance, baseline shift, and reduced detection and imaging resolution. The ion migration effect of an X‐ray detector is especially obvious at high driving voltage. To weaken the migration effect, an effective approach is to use MA_3_Bi_2_I_9_ SCs with a 0D structure as the X‐ray absorber, as proposed by Liu et al.^[^ ^57^ ^]^ The superior lead‐free SC was obtained at inch scale with high quality. Compared with other perovskites, the ion mobility and dark current of the 0D crystals is lower. Also it has better environmental stability. These properties are helpful for the performance of a perovskite X‐ray detector with the symmetric vertical‐type configuration (**Figure** **18**a). The sensitivity is as high as 1947 µC Gy_air_^−1^ cm^−2^, baseline drift as low as 5.0 × 10^−10^ nA cm^−1^ S^−1^ V^−1^, detection limit as low as 83 nGy_air_ s^−1^ and with fast responsivity (Figure 18b–f), which represents the best‐performing perovskite with medium and long‐term stability reported to date. Furthermore, an X‐ray imaging device using the 0D structure with excellent irradiation responsivity and superior crystal size was manufactured for the first time. Zhuang et al. fabricated a large‐area, low‐cost flat panel detector device using (NH_4_)_3_Bi_2_I_9_ SC with high sensitivity to X‐rays and an extremely low detection limit (Figure 18g–i).^[^ ^106^ ^]^ The absorber SC is easy to grow at low temperature and is easily cut based on cleavage planes. In addition, Zhang et al. further confirmed the ultra‐low ion mobility of 0D‐structured HP MA_3_Bi_2_I_9_ SC, and based on such crystals achieved excellent X‐ray detection stability and low detection limit.^[^ ^109^ ^]^ More recently, Liu et al. reported a centimeter‐scale all inorganic Cs_3_Bi_2_I_9_ SC for X‐ray detection with a sensitivity of 1.65 × 10^3^ µC Gy^−1^cm^−2^ at 60 V, with a LoD value up to 1.3 × 10^−1^ Gy s^−1^, which is consistent with the values of most inorganic lead halide detectors.^[^ ^88^ ^]^

#### 4.3.2 Co‐Planar Structure

**Figure 19:**
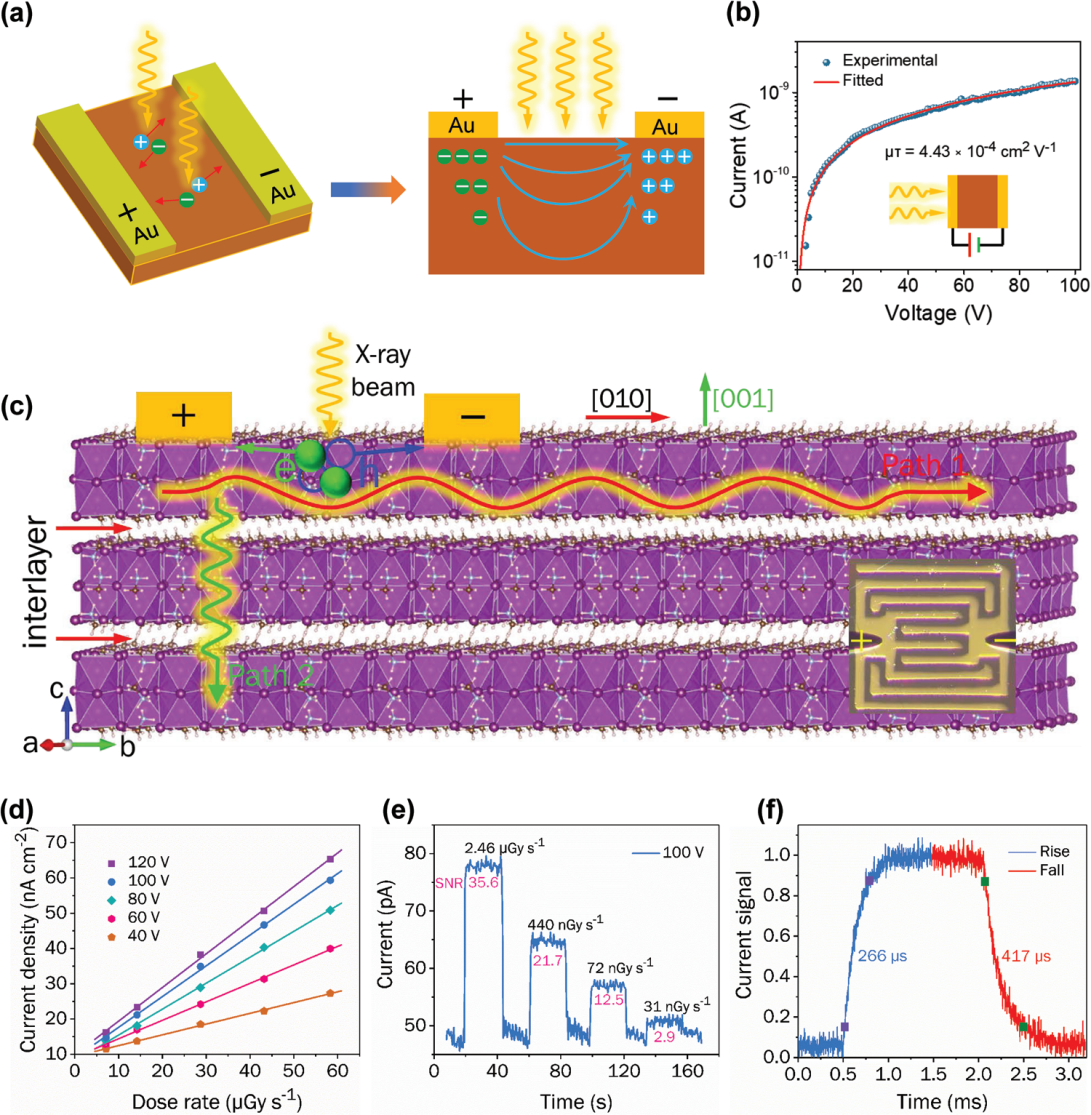
The response of an HP SC coplanar detector under X‐ray illumination. a) Schematic diagram of the coplanar‐structured detector and the corresponding electric field distribution in the device. b) Photoconductivity response of the (BDA)PbI_4_ SC. Reproduced with permission.^[^ ^166^ ^]^ Copyright 2022, Royalty Society of Chemistry. c) The carrier transport in MA_3_Bi_2_I_9_ SC along different directions parallel or perpendicular to the inorganic layer. d) Response current under different X‐ray dose rates and different bias voltages. e) Response current curve of the MA_3_Bi_2_I_9_ co‐planar X‐ray detector under various X‐ray dose rates and 100‐V bias voltage. f) Transient response curve of the MA_3_Bi_2_I_9_ SC coplanar detector under X‐ray illumination. Reproduced with permission.^[^ ^116^ ^]^ Copyright 2020, John Wiley and Sons.

The co‐planar‐structured HP SC detectors show a nonlinear as well as non‐uniform electric field.^[^ ^166^ ^]^ As shown in **Figure** **19**a, the electric field strength in the deeper region of the device is greatly reduced. Obviously, the distortion of the electric field exists in the planar device near the collecting electrode. This electric field distortion results in a low efficiency of charge collection, especially for the photogenerated electrons and holes in the deeper regions with smaller electric field. Theoretically speaking, the collected charges are mainly photogenerated carriers at the top surface, where the strength of the electric field is larger, resulting in a higher carrier collection efficiency. Therefore, the detectors with co‐planar‐structured electrodes should operate as surface‐sensitive detectors, which are suitable to achieve fast detection for low‐energy X‐ray photons. For detectors, charge collection is determined by the carrier mobility and *µτ*. Under working conditions, the *µτ* of SC can be obtained by using light conductivity measurement (Figure 19b).

In 2020, Liu et al. proposed a better inch‐sized (34.3 mm × 34.3 mm × 13.1 mm) high‐quality lead‐free halide material, (CH_3_NH_3_)_3_Bi_2_I_9_ single crystal (MA_3_Bi_2_I_9_ SC), which possesses higher resistivity, large ion activate energy, lower ion migration possibility and better environmental stability.^[^ ^116^ ^]^ The high X‐ray absorptivity of this material makes it more suitable for coplanar detectors. Therefore, they prepared a MA_3_Bi_2_I_9_ SC X‐ray detector in a co‐planar configuration. Compared with the vertical‐structured device, the main merit of the co‐planar‐structured device is that it disconnects the sensitivity from the carrier drift distance (Figure 19c), such that the CCE is insensitive to the thickness of the photon absorption material. That will markedly reduce the requirement for uniformity of the thick perovskite SC along the vertical direction, thus simplifying the device manufacturing process. As seen in Figure 19d, the co‐planar‐structured X‐ray detector made of MA_3_Bi_2_I_9_ SC delivered a small dark noise current and excellent response under X‐ray illumination, with a sensitivity of 872 µC Gy^−1^cm^−2^, a low X‐ray detection limit of 31 nGy s^−1^ (Figure 19e), a short response time of 266 µs (Figure 19f), as well as long‐term working stability.^[^ ^116^ ^]^ Because of these excellent characteristics, the co‐planar structural X‐ray detector made of MA_3_Bi_2_I_9_ SC can distinguish the size, shape, thickness, and other characteristics of various objects and display high‐resolution X‐ray imaging. In order to summarized the X‐ray detection performance of the vertical‐structure and the co‐planar structure detectors, we compared their key parameters in **Table** **2**.

**Table 2:** Performance comparison of direct X‐ray detectors based on perovskite SC with vertical (V) and co‐planar (P) structures

Materials	Structure [V or P]	µ*τ* product [cm^2^ V^−1^]	Electric field [V mm^−1^]	Sensitivity [µC Gy^−1^ cm^−2^] [kV]	Detection limit [nGy s^−1^]	Refs.
MAPbBr_3_ SC	V	1.2 × 10^−2^	15	80(50)	≈500	[71]
MAPbBr_3_ SC	V	2.6 × 10^−4^	50	≈529	1210	[74]
MAPbI_3_ film	V	2.0 × 10^−7^	‐	‐	‐	[79]
MAPbI_3_ film	V	1.0 × 10^−4^	2400	11 000(100)	‐	[35]
MAPbI_3_ SC	V	3.26 × 10^−3^	≈10	968.9	‐	[80]
MAPbI_3_ SC	V	‐	15	21 000(60)	18000	[82]
MAPbI_3_ Wafer	V	2.0 × 10^−4^	2000	2527(70)	48000	[83]
CsPbBr_3_ SC	V	2.5 × 10^−3^	20	1256(80)	‐	[95]
CsPbBr_3_ film	V	1.32 × 10^−2^	50	55 684	215	[97]
Cs_3_Bi_2_I_9_ SC	V	7.97 × 10^−4^	50	1652.3	130	[101]
Cs_2_AgBiBr_6_ SC	V	‐	6	316	‐	[21]
(DMA)MAPbI_3_ SC	V	‐	4.2	12 000(8)	16.9	[91]
(GA)MAPbI_3_ SC	V	‐	4.2	23 000(8)	16.9	[91]
Polymer‐Encapsulated Cs_4_PbI_6_ Thin Film	V	‐	‐	256.20(30)	‐	[121]
MAPbBr_2.94_Cl_0.06_ SC	V	1.8 × 10^−2^	600	84 000	7.6	[76]
Cs_2_AgBiBr_6_ SC	V	6.3 × 10^−3^	‐	105(50)	59.7	[55]
MA_3_Bi_2_I_9_ SC	V	2.87 × 10^−3^	50	1947(40)	83	[57]
(NH_4_)_3_Bi_2_I_9_ SC	V	1.1 × 10^−2^	10	8200(50)	55	[106]
MA_3_Bi_2_I_9_ SC	P	‐	28 600	872	31	[116]
MAPbI_3_ SC	P	‐	1000	5.2 × 10^6^(50)	0.1	[167]
MAPbI_3_ SC	P	‐	100	≈7 × 10^5^ (50)	1.5	[81]
(DMEDA)BiI_5_ SC	P	‐	494	72 500(50)	‐	[114]
(DMEDA)BiI_5_ SC	P	‐	7.24	1600(50)	‐	[114]
BDAPbI_4_ SC	P	4.43 × 10^−4^	494	242(40)	430	[115]
Rb_3_Bi_2_I_9_ SC	P	2.51 × 10^−3^	2000	159.7(50)	8.3	[111]
MAPbBr_3_ SC Film	P	1.2 × 10^−2^	‐	80	500	[6]
CsPbBr_3_ Film	P	‐	‐	1450	17.2 × 10^3^	[6]

#### 4.3.3 Heterojunction Structures of HP for X‐Ray Detectors

**Figure 20:**
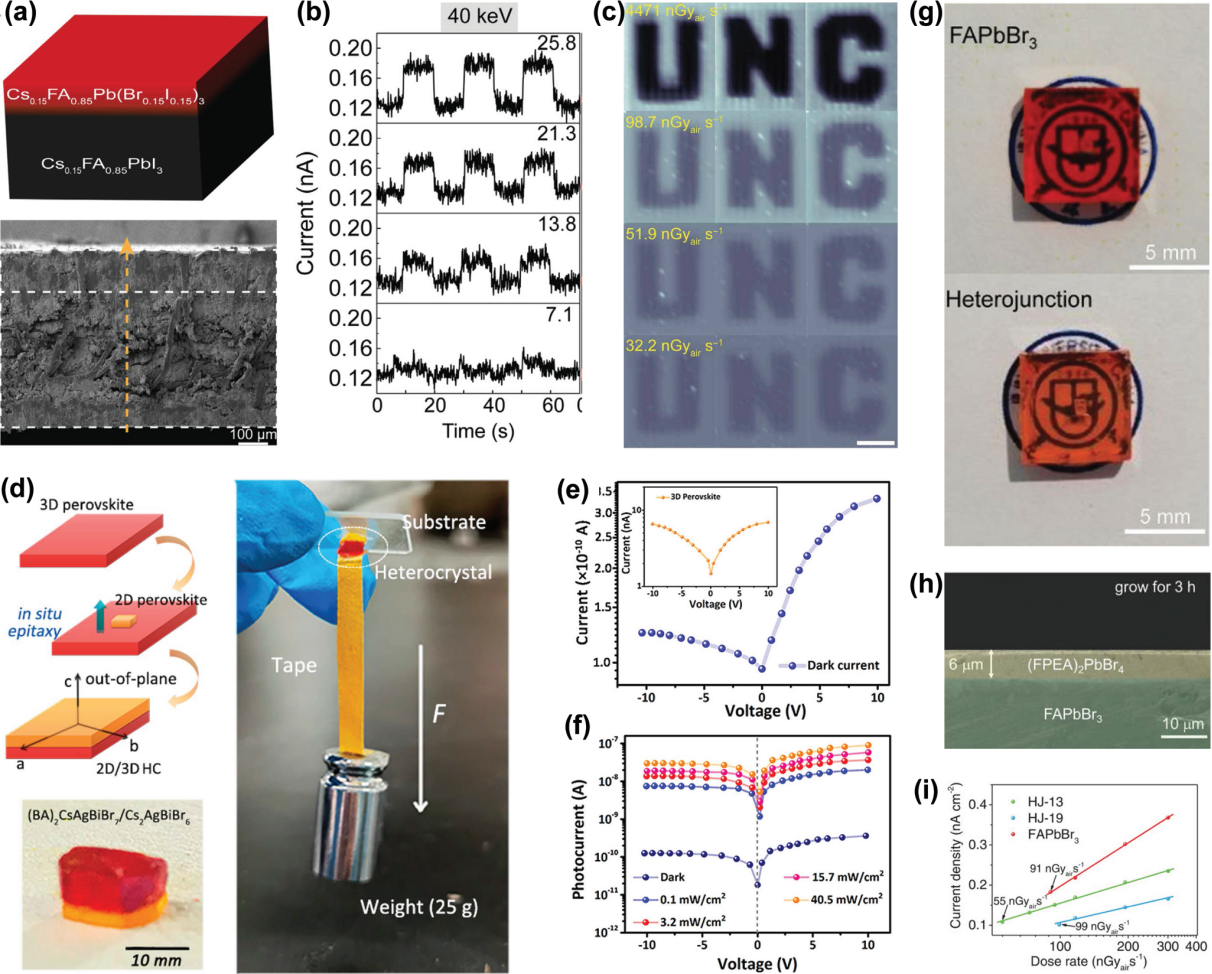
Perovskite heterojunction and corresponding detector devices. a) Schematic illustration of the 3D perovskite heterojunction of Cs_0.15_FA_0.85_PbI_3_ and Cs_0.15_FA_0.85_Pb(I_0.15_Br_0.85_)_3_. b) The current response of the heterojunction device to the chopped 40‐keV X‐ray source with variable dose rates under an electric bias of ‐25 V. c) X‐ray images of the UNC logo obtained by the heterojunction detector under different X‐ray fluxes. Reproduced with permission.^[^ ^90^ ^]^ Copyright 2021, AAAS. d) Photograph of the (BA)_2_CsAgBiBr_7_/Cs_2_AgBiBr_6_ heterocrystal. e) *I–V* curves of the heterocrystal device measured in the dark. f) *I–V* curves of the heterocrystal device under different light intensities of a 405‐nm laser. Reproduced with permission.^[^ ^5^ ^]^ Copyright 2021, American Chemical Society. g) Photograph of a FAPbBr_3_ SC. h) Photograph of a FAPbBr_3_/(FPEA)_2_PbBr_4_ 3D/2D heterojunction SC. i) Current density dependence on X‐ray dose rate of the SC detectors. Reproduced with permission.^[^ ^169^ ^]^ Copyright 2021, John Wiley and Sons.

Perovskite polycrystalline thin films are good candidates for X‐ray detection applications due to their excellent X‐ray absorption coefficient, good charge carrier transport, and solution processability. But the large leakage current under dark conditions has a negative impact on the quality of X‐ray images under low‐dose conditions. HP heterostructures, including Schottky heterostructures, type II heterostructures and z‐heterostructures, can improve carrier separation, accelerate the surface reaction rate and achieve efficient energy conversion.^[^ ^168^ ^]^ The design and fabrication of perovskite heterojunctions is an effective way to reduce the low detection limit of X‐ray PDUs, so as to reduce the X‐ray dose during the imaging process. Zhou et al. significantly reduced the leakage current by designing and constructing a heterojunction.^[^ ^90^ ^]^ Two porous flexible Nylon films were separately immersed into the precursor solution of Cs_0.15_FA_0.85_PbI_3_ and Cs_0.15_FA_0.85_Pb(I_0.15_Br_0.85_)_3_ and further calcined and stamped together at high temperature. Since the band structures (the energy positions of the valence band maximum (VBM) and conduction band minimum (CBM)) of these two perovskite materials are different, a heterojunction was formed (**Figure** **20**a). The heterojunction device can markedly reduce the leakage current while keeping the high sensitivity to X‐ray photons. It can be seen from Figure 20b,c that the detector has a minimum detection X‐ray dose rate as small as 13.8 ± 0.29 nGy_air_ s^−1^ for 40‐keV X‐rays, can achieve clear X‐ray imaging at a low X‐ray photon flux with dose rate of 32.2 nGy_air_ s^−1^, under which the characters “UNC” can be clearly identified. 2D perovskite can effectively suppress or eliminate the ion migration, while 3D perovskite materials show higher sensitivity to X‐ray photons. The 2D and 3D perovskite heterojunction can achieve the best performance combining the merits of these two types of materials, thereby reducing the detection limit dose rate and maintaining the high sensitivity to X‐ray photons. Zhang et al. synthesized advanced 2D and 3D perovskite, and grew high‐quality heterocrystals of lead‐free HP 2D/3D (BA)_2_CsAgBiBr_7_ /Cs_2_AgBiBr_6_, with a macroscopic size (cm) (Figure 20d).^[^ ^5^ ^]^ Heterogeneous crystal X‐ray detectors can work in a self‐driving mode and achieve an amazing X‐ray sensitivity of 206 µC Gy^−1^ cm^−2^, which is superior to the pristine Cs_2_AgBiBr_6_ SC detector, with an ultra‐low leakage current of 3.2 × 10^−2^ pA under high bias as well as remarkable working stability (Figure 20e,f). He et al. used long organic cations to replace short formamide (FA^+^) ions, and obtained a large‐size 3D/2D perovskite heterojunction. The defect density of the heterojunction is as low as 3.18 × 10^9^ cm^−3^, and its carrier mobility (80.43 cm^2^ V^−1^ s^−1^) is higher than that of the 3D perovskite Sc (Figure 20g–i).^[^ ^169^ ^]^

In addition, Nie et al. designed a new type of p‐i‐n junction thin‐film device, which used 2D Ruddlesden‐Popper (RP) phase layered perovskite (BA)_2_(MA) _2_Pb_3_I_10_ to detect X‐ray photons efficiently.^[^ ^112^ ^]^ Its high diode resistivity of 10^12^ Ω cm under reverse bias leads to an X‐ray detection sensitivity as high as 0.276 C Gy_air_^−1^ cm^−3^. This large signal acquisition is realized by the potential underpinning operation built in the original photocurrent device, which is robust. The detector generates a large number of X‐ray photons to induce an open‐circuit voltage, which provides an alternative detection mechanism. He et al. prepared a quasi‐2D perovskite thick film by inserting a 2D Ruddlesden‐Popper perovskite layer into the 3D perovskite film to suppress ion migration. The sensitivity of this device reached 10860 µC Gy_air_^−1^cm^−2^. The detector also shows a stable dark current and response current. The low detection limit of 69 nGy_air_ s^−1^ was the lowest among all reported polycrystalline thin‐film detectors.

### 4.4 Halide Perovskites for X‐Ray Imaging

**Figure 21:**
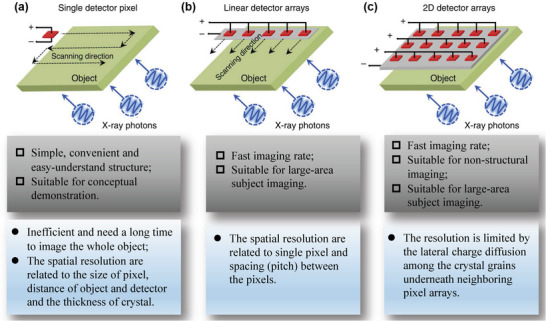
Schematic diagrams of imaging modes and features of three different kinds of X‐ray imaging processes: a) single pixel detection, b) 1D detector array scanning, c) 2D detector arrays coupled with DAC timer scanning. The different characteristics (pros and cons) of the three different imaging processes are listed below them. Reproduced with permission.^[^ ^15^ ^]^ Copyright 2019, Springer Nature.

In terms of imaging, a low‐temperature fabrication process is the key factor for depositing semiconductors onto a‐Se thin film transistors, which will be destroyed under high temperature exceeding 200 °C. In addition to the process temperature, the physical connection between the deposited semiconductor active layer and the a‐Si substrate should be manipulated to reduce the possibility of film peeling and damages related to the optical coupling. The signal at each pixel should be uniform without image lag.^[^ ^166^ ^]^ These issues representative many challenges of traditional X‐ray imaging materials. Currently, there are three kinds of imaging configurations: single detector pixel (**Figure** **21**a), linear detector array (Figure 21b) and 2D detector array (Figure 21c). Figure 21 lists the advantages and disadvantages of the three imaging configurations. In this section, we will focus on some excellent works regarding X‐ray imaging devices utilizing HPs.

#### 4.4.1 Single Pixel Detector for Imaging by Point‐to‐Point Scanning

**Figure 22:**
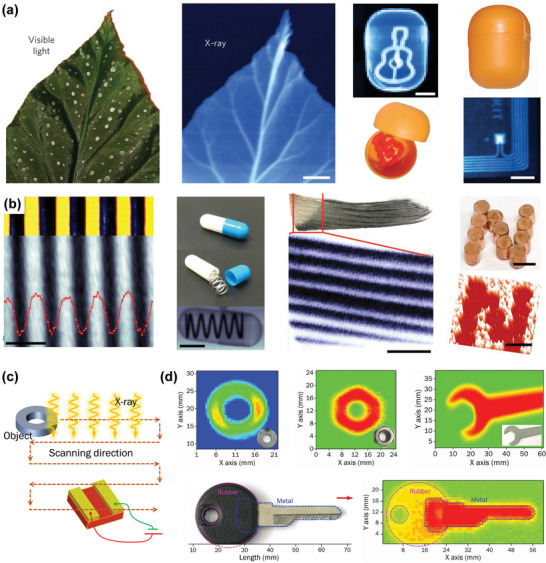
Single‐pixel HP X‐ray detector for imaging. a) Optical images and corresponding X‐ray images of a leaf, a Kinder egg and an electronic key card with a chip and integrated radio‐frequency antenna. Reproduced with permission.^[^ ^79^ ^]^ Copyright 2019, Springer Nature. b) Optical image (top) and X‐ray image (bottom) of a stainless‐steel plate that has lines etched through, a spring inside a capsule, a part of a fish fin and an “N”. Reproduced with permission.^[^ ^70^ ^]^ Copyright 2017, Springer Nature. c) Schematic illustration of single‐pixel detector scanning‐mode X‐ray imaging. The X‐ray images of objects with different shapes and thicknesses obtained using the MA_3_Bi_2_I_9_ SC coplanar X‐ray detector. d) a 1 mm‐thick metal gasket, a 5 mm‐thick nut, a 2 mm‐thick wrench and a key with a junction between the metal part and opaque rubber. Insets: the corresponding photographs of the objects. Reproduced with permission.^[^ ^57^ ^]^ Copyright 2020, Elsevier.

For a single pixel detector, its spatial resolution is determined by the active size of the detector, the distance between the object and the detector, and the sensitivity of the detector. Generally, in commercial imaging, the size of a pixel should be <10 µm. The smaller is the pixel size, the larger is the crosstalk from the adjacent pixels. Furthermore, the optoelectronic coupling between the photon‐sensitive semiconductor and substrate has a strong relationship with the imaging clarity. In 2015, Yakunin et al. prepared a p‐i‐n diode X‐ray detector with MAPbI_3_ polycrystalline film for the first time, and measured its suitability for imaging using a single‐pixel photoconductor in the imaging process. X‐ray beams scanned various objects in 2D, as shown in **Figure** **22**a.^[^ ^79^ ^]^ Several images with good resolution were obtained: the leave of an indoor plant leaf, a kinder surprise egg and an electronic card. In all the images, the details inside the object have been detected and presented, which cannot be seen from the outside. The obtained images show high contrast and dynamic range, and their spatial resolution is only limited by the size of the photodetectors and X‐ray beam collimation. Subsequently, Wei et al. proposed a strategy to directly grow MAPbBr_3_ SC on a Si substrate, between which strong mechanical adhesion was achieved by brominating (3‐aminopropyl) triethoxysilane molecules (APTES) as a connecting layer.^[^ ^70^ ^]^ The organic group of Si‐O‐C_2_H_5_ in APTES tended to hydrolyze, which will result in the formation of Si‐OH group. The group can bond tightly with the —OH group at the Si surface, leading to the formation of a strong covalent Si‐O‐Si bond. Meanwhile, NH_3_Br was formed by reaction between the —NH_2_ group and HBr, which induced the formation of MAPbBr_3_ SC. Finally, an X‐ray detector with the device structure of Si/MAPbBr_3_/C_60_/BCP/Au was assembled and used for single‐pixel scanning imaging. The system was able to distinguish objects <80 µm and achieved a spatial resolution of ≈10 lp mm^−1^ (Figure 22b).^[^ ^79^ ^]^

In addition, Liu's team also formed lead‐free MA_3_Bi_2_I_9_ SC and used it to manufacture coplanar X‐ray detectors.^[^ ^116^ ^]^ The capability of a single‐pixel detector in X‐ray imaging was evaluated. Small objects with different thicknesses and shapes were loaded onto an *X–Y* scanning table. The X‐ray images were constructed by plotting the 2D position‐related response current values, which were recorded by moving the object in the *X–Y* plane between the X‐ray source and the detector and digitalized by the software as *I_x‐‐y_* (Figure 22c). As shown in Figure 22d, images of a small metal gasket, a nut and a spanner can be obtained with good spatial resolution. The X‐ray images show sufficient contrast, indicating the MA_3_Bi_2_I_9_ SC X‐ray detector can be used to distinguish different object features like physical size, shape, and thickness.

#### 4.4.2 Linear Array Imaging

**Figure 23:**
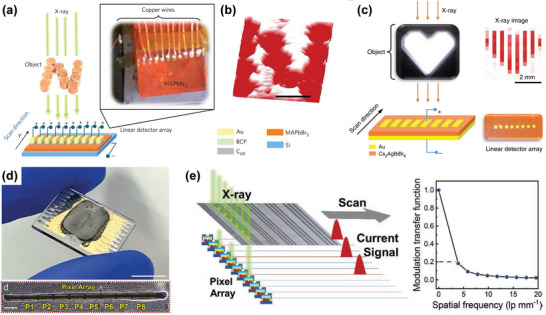
Linear‐array HP X‐ray detectors for imaging. a) Schematic illustration of the X‐ray imaging device with Si‐integrated MAPbBr_3_ SC as the active material of X‐ray detectors. b) X‐ray image of an “N” detected using the 1D linear detector array. Reproduced with permission.^[^ ^70^ ^]^ Copyright 2021, John Wiley and Sons. c) Optical image and X‐ray image of a heart‐shaped object obtained using the BiOBr‐passivated Cs_2_AgBiBr_6_ wafer linear X‐ray detector array. Reproduced with permission.^[^ ^104^ ^]^ Copyright 2019, Springer Nature. d) Photograph and enlarged view, and e) spatial resolution of the MAPbI_3_ SC‐based coplanar X‐ray detector linear array. Reproduced with permission.^[^ ^167^ ^]^ Copyright 2021, John Wiley and Sons.

A 1D linear detector array is simply composed of X‐ray detectors arranged in a line. Different from the single pixel detector, the spatial resolution of the linear pixel array detector depends not only on the size of a single pixel, but also on the distance between neighboring pixels. Wei et al. made a 10‐pixel linear array imaging detector using MAPbBr_3_ SC.^[^ ^70^ ^]^ As shown in **Figure** **23**a. Each pixel has a separate copper wire integrated with the current amplifier, and the doped Si wafer acts as an anode. During the imaging, an “N” consisting of copper pieces was inserted between the X‐ray source and the X‐ray detector array. As the subject was moved, position‐related electrical signals were measured and recorded by the data acquisition system, with the result shown in Figure 23b. Then, Yang et al. prepared large‐area Cs_2_AgBiBr_6_ wafers by isostatic pressing and included a heteroepitaxially grown BiOBr layer to passivate the defects at grain boundaries, inhibiting the dangling chemical bonds, and preventing ion migration.^[^ ^170^ ^]^ As the BiOBr layer can reduce the leakage current and result in uniform photocurrent response, a spatial resolution of 4.9 lp mm^−1^ was obtained, and the imaging obtained by this X‐ray detector array is showing in Figure 23c.^[^ ^104^ ^]^ Dong et al. assembled a linear detector array on MAPbI_3_ SC showing excellent X‐ray imaging capability (Figure 23d);^[^ ^167^ ^]^ however, the spatial resolution of the device (Figure 23e) is much lower than that required by commercial standards of medical imaging, and it needs to be increased more than three times to meet the medical requirement (resolution of 10 lp mm^−1^).

#### 4.4.3 2D Array Imaging

**Figure 24:**
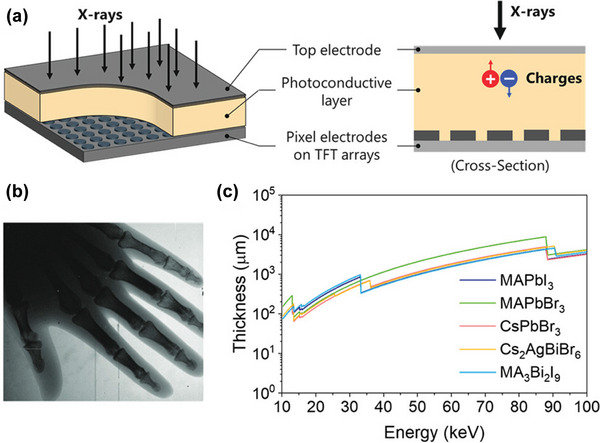
The application of perovskite materials in the FPD imaging configuration. a) Schematic illustration of a direct FPD with a thick semiconductor layer as the charge generation layer and pixel TFT array and top electrode for charge collection, which have been summarized in the cross‐sectional device structure. b) The X‐ray image of a hand phantom obtained by a Pe‐FPD. c) The estimated thickness for various perovskite materials to absorb 98% of the flux of 10–100 keV X‐rays. Reproduced with permission.^[^ ^8^ ^]^ Copyright 2022, American Chemical Society.

In order to realize a perovskite‐based flat panel detector (Pe‐FPD), a large‐area perovskite layer must be prepared on a thin film transistor (TFT) array for direct X‐ray FPD applications. **Figure** **24**a shows the normal device structure of a TFT‐based direct FPD. The absorber layer (perovskite) is deposited onto the TFT array and covered with a top electrode.^[^ ^8^ ^]^ The X‐rays penetrating the imaging target are absorbed by the calcite layer, which can convert the X‐ray photons to electric charges. The generated charge is directly collected by the pixel electrode under an electric field, which has been applied between the upper electrode and the lower electrode. The difference in the amount of charge collected from each pixel will generate contrast; then an X‐ray image can be constructed (Figure 24b). In a word, the calcite layer plays an important role in the absorption of X‐rays photons as well as the transport and collection of charges. According to this basic working mechanism, the requirements for the preparation process of the perovskite layer for FPD application are as follows: medical imaging, mammography, and angiography generally require 24 × 30 and 30 × 40 cm^2^ large detection areas. The thickness of the absorber layer should fully absorb X‐ray photons and effectively transmit carriers with a small loss. As shown in Figure 24c, depending on the X‐ray energy used in the application and the material's attenuation coefficients to X‐ray photons, 100–5000 µm thickness is needed.

## 5 Advancements in HP Indirect‐Type X‐Ray Detectors

Generally speaking, the indirect‐type X‐ray detector utilizes a scintillator to convert X‐ray photons to UV–vis photons. HP scintillators possesses high PLQYs, tunable optical bandgap and emission peaks, and short afterglow time, resulting in outstanding scintillation characteristics, such as high LY values, tunable radiation peaks and response time in the nanosecond region. So far, HP materials with high emission intensity covering the whole visible region have been developed. Among those HP materials, the LY at room temperature is 640 00 photons MeV^−1^, outperforming the current inorganic scintillator. When the temperature is reduced to 10 K, the LY of MAPbI_3_ single crystal can reach as high as 300 000 photons MeV^−1^. In addition, the lead‐free HP and perovskite‐like materials have outstanding scintillation characteristics. For example, the Rb_2_CuBr_3_ scintillator shows very high LY of ≈91 056 photons MeV^−1^ at room temperature.

### 5.1 HP Nanocrystal and Quantum Dots Scintillators

#### 5.1.1 HP Nanocrystal Scintillator

**Figure 25:**
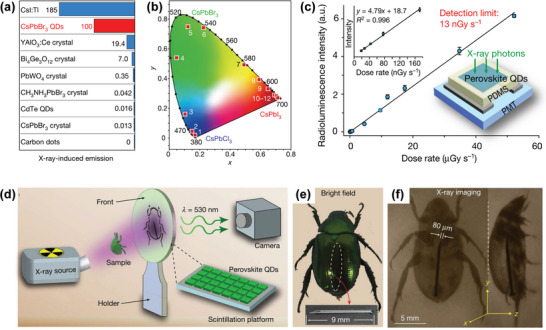
The properties of CsPbX3 NCs‐based X‐ray scintillation materials: a) Summary of the optical sensitivity of various scintillator materials under exposure to X‐rays. b) CIE chromaticity coordinates of the X‐ray‐induced visible emissions of samples 1–12. c) The dose rate‐dependent radioluminescence intensity of a CsPbBr_3_ scintillator. The detection limit to X‐rays of 13 nGy s^−1^ is estimated from the slope of the fitting line, with a signal‐to‐noise ratio of 3. d) Schematic illustration of the experimental setup for real‐time X‐ray imaging. A beetle is used as the object, which was mounted onto a plate between the scintillation film and X‐ray source. E) Bright‐field picture and f) the 50‐keV X‐ray‐induced picture under dark conditions of the sample, which were obtained with a camera. Reproduced with permission.^[^ ^53^ ^]^ Copyright 2018, Springer Nature.

Nanocrystals (NCs) or quantum dots (QDs) are promising candidates for scintillation applications as they exhibit excellent scintillation behavior at room temperature.^[^ ^156^ ^]^ CsPbBr_3_ NCs have also been applied to the assembly of scintillation films.^[^ ^9^ ^]^ Due to their high exciton binding energy and the large quantum confinement of the small NCs, they usually show high PLQYs and emission peaks with small full‐width at half‐maximum (FWHM). Therefore, perovskite NCs are thought to be great candidates for use in lasers and LEDs, but their applications in photodetectors rarely have been investigated. In 2015, high‐quality CsPbX_3_ (X = I^−^, Br^−^, Cl^−^, or mixed halide) NCs with PLQYs up to 90% were synthesized by the thermal injection method. Ramasamy et al. showed a photodetector device based on all‐inorganic CsPbI_3_ NCs for the first time.^[^ ^171^ ^]^ They synthesized CsPbI_3_ NCs by heat injection, and then added lithium salt (LiCl or LiI) to obtain CsPbCl_3_ and CsPbI_3_ nanocrystals by halide exchange reaction. The group further reported a hybrid photodetector based on graphene‐CsPbBr_3_ *_x_* I*_x_* NCs,^[^ ^172^ ^]^ which showed high sensitivity. Dong et al. prepared a photodetector of solution‐treated high‐performance nanocrystalline CsPbBr_3_ that showed a preferred film orientation by centrifugal casting.^[^ ^173^ ^]^ The Au plasma effect further improved the detector's performance. Jang et al. reported an intriguing strategy synthesize of APbX_3_ perovskite NCs, where A = CH_3_NH^3+^, Cs^+^, or CH(NH_2_)^2+^, and X = Cl, Br, or I. by ultrasonic treatment.^[^ ^174^ ^]^ Chen et al. reported polychromatic Xray scintillators prepared from a series of all‐inorganic CsPbX_3_ nanocrystals, as shown in **Figure** **25**a,b.^[^ ^53^ ^]^ Unlike the other kinds of inorganic scintillators, perovskite nanomaterials can be solution‐prepared, modified, and treated at low temperatures, and can easily be adjusted to achieve X‐ray‐induced emission covering the whole visible spectrum by customizing the halide ion components of the colloidal precursors during the synthesis. In general, the X‐ray‐induced signal of the spin‐coated CsPbBr_3_ nanocrystalline film has good linearity in a wide range of X‐ray dose rates (Figure 25c), and delivers a detection limit as small as 13 nGy s^−1^, which is lower than that of conventional medical diagnosis (5.5 µGy s^−1^) by a factor of ≈420. At the same time, the scintillator has a fast response time (44.6 ns) under the illumination of a Cs_137_ source (661 keV), making it an ideal choice for indirect scintillation imaging. In order to achieve good imaging performance from the perovskite scintillators, they integrated an aluminum foil protective cover and CsPbBr_3_ nanocrystalline film onto an *α*‐Si photodiode array and a TFT readout unit to assemble the X‐ray imaging system, which can be directly used for digital radiographic imaging (Figure 25d–f). The modulation transfer function (MTF) value of this detector at the spatial resolution of 2.0 lp mm^−1^ is as high as 0.72, which is higher than that of the widely used CsI:Tl‐based detector (0.36 at 2.0 lp mm^−1^). The high spatial resolution and low detection limit of CsPbBr_3_ nanocrystalline thin‐film scintillators enables them to create high‐quality X‐ray images at 15 µGy X‐ray dose, a relatively low dose for X‐ray imaging.

#### 5.1.2 HP QDs Scintillator

HP QDs are thought to have good integration characteristics of quantum size effect, enhanced optical properties compared with their 3D bulk counterparts, tunable surface chemistry, and a “free” colloidal state, which makes them dispersible in various organic solvents and matrices, and finally, they are easily integrated into various devices.^[^ ^175^ ^]^ Liu et al. proposed a reasonably priced inkjet‐printing method to prepare a large‐area uniform colloidal CsPbBr_3_ quantum dot film for use in an X‐ray detector.^[^ ^6^ ^]^ At a 0.1 V bias voltage, the detector showed distinct response current to a very low X‐ray flux with a dose rate of 17.2 mGy_air_ s^−1^, while showing a high X‐ray sensitivity.^[^ ^176^ ^]^ However, the environmental instability of perovskite QDs as well as the strong light scattering of a thick, opaque QDs scintillator film hinder them from obtaining high‐resolution and robust X‐ray images. Recently, Yang et al. reported europium (Eu)‐doped CsPbBr_3_ quantum dot glass‐ceramic scintillators with a spatial resolution of 15.0 lp mm^−1^.^[^ ^177^ ^]^

#### 5.1.3 Combination of HP Materials

**Figure 26:**
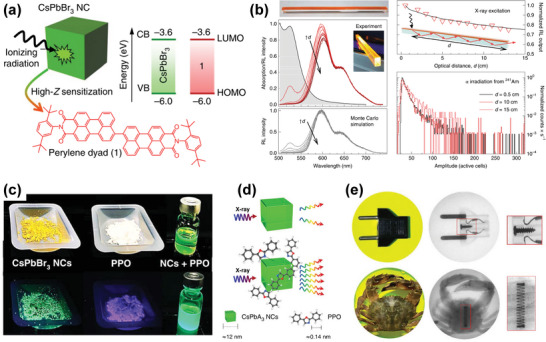
a) Schematic diagram of the CsPbBr_3_ nanocrystal sensitizing the perylene dyad. b) Waveguiding tests on CsPbBr_3_ nanocrystal‐sensitized plastic scintillators. Reproduced with permission.^[^ ^50^ ^]^ Copyright 2020, Springer Nature. c) Photographs of CsPbBr_3_ nanocrystals, PPO, and colloidal hybrid CsPbBr_3_ nanocrystals +PPO in octane under illumination with white and UV light. d) Schematic illustration of the radiation luminescence of the PPO hybridized CsPbA_3_ NC and CsPbA_3_ NC. e) Optical and X‐ray images of an electric power plug and a crab. Reproduced with permission.^[^ ^179^ ^]^ Copyright 2020, Springer Nature.

HP NCs are promising for X‐ray imaging at room temperature due to their impressive scintillation performance;^[^ ^178^ ^]^ however, the problem is that their stability to X‐ray illumination is poor, not only due to their inherent high chemical reactivity with a variety of environmental species, light illumination induced halide migration, and instability under exposure to heat and water, but also because of their aggregation properties and phase transition problems. Therefore, HPs QDs must be modified or tuned to achieve high scintillation performance as well as reliable stability under X‐ray irradiation. To achieve the final scintillation film, NCs have always been immersed in polymer materials; however, a high weight ratio of polymer content can significantly impair the LY due to its small radiation absorption properties. By amplifying a Stokes shift to separate the absorption spectrum from the emission spectrum, this problem should be solved due to the transparency of the scintillator to the photons it emitted. Recently, Gandini et al. reduced the self‐absorption problem by combining CsPbBr_3_ nanocrystals with conjugated organic dyes, which can absorb the light emitted by CsPbBr_3_ nanocrystals and emit photons unabsorbed by the CsPbBr_3_ NCs, as shown in **Figure** **26**a.^[^ ^50^ ^]^ The organic molecule used in this composite is perylene dicarboxylate (i.e., 9,9 '‐ bis [perylene‐3,4‐dicarboxyl‐3,4 – (*N*‐(2,5‐di‐tert‐butylphenyl)], whose highest occupied molecular orbital (HOMO) and lowest unoccupied molecular orbit (LUMO) energy levels match well with the valence band maximum and conduction band minimum of CsPbBr_3_ nanocrystals. The composite film showed a large Stokes shift and formed a plastic scintillator with almost no reabsorption (Figure 26b). However, its LY is as small as 9000 photons MeV^−1^, which is much lower than that of pure CsPbBr_3_ NCs film. Cho et al. developed a liquid scintillator composed of CsPbX_3_ nanocrystals hybridized with the surface of 2,5‐diphenyloxycloxazole (PPO), as shown in Figure 26c,d.^[^ ^179^ ^]^ In general, PPO^+^ and CsPbBr_3_ liquid phase scintillators have good X‐ray imaging performance, with a high spatial resolution of 3.5 lp mm^−1^, thus achieving high‐spatial‐resolution imaging (Figure 26e). Heo et al. developed a scintillator film based on CsPbBr_3_ nanocrystals composed of a carbon fiber‐enhanced polymer film/scintillator film/Si‐PD array.^[^ ^48^ ^]^ The scintillator thin film is assembled by photopolymerization of CsPbBr_3_ NCs, a photo‐initiator and methyl methacrylate. The thickness of the scintillator thin film can be tuned by adjusting the precursor solution concentration and spin‐coating speed. The conversion efficiency of the CsPbBr_3_ nanocrystalline film is 45–49%, which is much higher than film (10–24%). The X‐ray image obtained by the CsPbBr_3_ nanocrystalline thin film detector has higher spatial resolution than that captured by GOS thin film, especially under low X‐ray dose.

Recently, Wu et al. prepared Ce^3+^‐doped CsPbBr_3_ nanocrystals with a thickness of 30 µm and transparent high‐density scintillator film.^[^ ^180^ ^]^ The scintillator thin films made of these high‐density nanomaterials, with their thin and transparent characteristics, have achieved a high resolution of 862 nm, which is the highest resolution for perovskite scintillator‐based X‐ray microscopic imaging so far. In addition, the PLQY of In^+^‐doped Cs_3_Cu_2_I_5_ also increased from 68.1 to 88.4%.^[^ ^181^ ^]^ Because of the higher PLQY, Cs_3_Cu_2_I_5_:In^+^ achieved an X‐ray detection limit as low as 96.2 nGy_air_ s^−1^. Furthermore, its scintillation yield of 53 000 photons MeV^−1^ is comparable to commercial single‐crystal CsI:Tl. As a result, a high spatial resolution of 18 lp mm^−1^ was realized. These results highlight the importance of efficiently capturing excitons of low‐dimensional perovskite in X‐ray detection applications.

**Figure 27:**
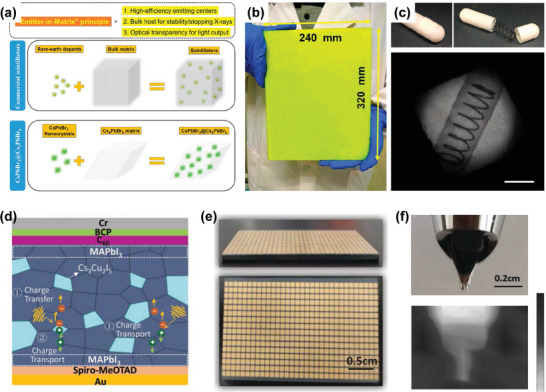
a) Illustration of the emitter‐in‐matrix CsPbBr_3_@Cs4PbBr_6_ structure. b) Large‐area CsPbBr_3_@Cs_4_PbBr_6_ film. c) Photograph and X‐ray image of a capsule containing a spring. Reproduced with permission.^[^ ^178^ ^]^ Copyright 2020, American Chemical Society. d) Mechanisms of two types of charge‐transport paths in hybrid devices. e) Photograph of a hybrid detector array. f) Photograph and X‐ray image of a pen nib. Reproduced with permission.^[^ ^183^ ^]^ Copyright 2022, John Wiley and Sons.

The combination of 0D and 3D perovskite materials (e.g., CsPbBr_3_@Cs_4_PbBr_6_), has been proved to be an ideal method to improve scintillation properties.^[^ ^178^ ^]^ The CsBr bridge wholly separates the [PbBr_6_]^4−^ octahedron in the Cs_4_PbBr_6_ structure and forms 0D perovskite, while the [PbBr_6_]^4−^ octahedron is connected in three directions to form 3D CsPbBr_3_. Although 3D HP nanomaterials have good prospects and have high scintillation performance even at room temperature, they are not stable under irradiation with high‐energy photons because they are sensitive to light, heat, humidity, and they undergo aggregation and phase transition. Therefore, it is still a difficult task to obtain stability and high scintillation efficiency under high‐energy X‐ray irradiation. The “matrix emitter” material can solve these problems. Cao et al. prepared a CsPbBr_3_@Cs_4_PbBr_6_ “Matrix emitter” structure to improve stability and scintillation performance (**Figure** **27**a) as the Cs_4_PbBr_6_ matrix can simultaneously passivate the highly active surfaces of CsPbBr_3_ nanocrystals and inhibit the aggregation of isolated CsPbBr_3_ nanocrystals, thus enhancing the stability under X‐ray radiation.^[^ ^178^ ^]^ In addition, they prepared large‐area CsPbBr_3_@Cs_4_PbBr_6_ film (36 × 24 cm^2^) by the blade‐coating method for an imaging screen, and then high‐performance X‐ray imaging was achieved (Figure 27b,c). Xu et al. prepared similar components of CsPbBr_3_/Cs_4_PbBr_6_, further demonstrating that CsPbBr_3_/Cs_4_PbBr_6_ showed excellent radiation hardness and high stability in air. The photo‐yield of CsPbBr_3_/Cs_4_PbBr_6_ is ≈64 000 photons MeV^−1^, ≈168% that of NaI:Tl and 156% that of CsI:Na.^[^ ^182^ ^]^ In addition, Liu et al. reported a new hybrid‐wafer X‐ray detector that combines the direct‐type MAPbI_3_ and the indirect‐type 0D Cs_3_Cu_2_I_5_ luminescent body through a low‐cost rapid tablet pressing process (Figure 27d–f).^[^ ^183^ ^]^ Because of the rapid energy transfer between Cs_3_Cu_2_I_5_ and MAPbI_3_, the response speed of the device to X‐rays is greatly shortened by ≈30 times, reaching 36.6 ns, so that the large‐area detector array has the capability of fast X‐ray detection within 1 s. In addition, Cs_3_Cu_2_I_5_ occurs at the grain boundary of MAPbI_3_ blocking the path of ion migration, so that the detection limit of this X‐ray detector is 1.5× smaller than that of the direct‐type MAPbI_3_ and 10× lower than that of the indirect‐type Cs_3_Cu_2_I_5_ scintillator. The direct/indirect hybrid wafers also show higher operational stability in the environment without packaging. Li et al. designed a scintillation screen with a CsPbBr_3_ nanocrystalline array pixel structure based on anodized aluminum oxide (AAO), and studied its scintillation characteristics for X‐ray imaging. It is worth noting that the pixelated CsPbBr_3_‐AAO array scintillation screen has successfully achieved a spatial resolution of 2 µm, short PL decay time of 9 ns and high stability exceeding 42 days.

### 5.2 Perovskite SC Scintillators

**Figure 28:**
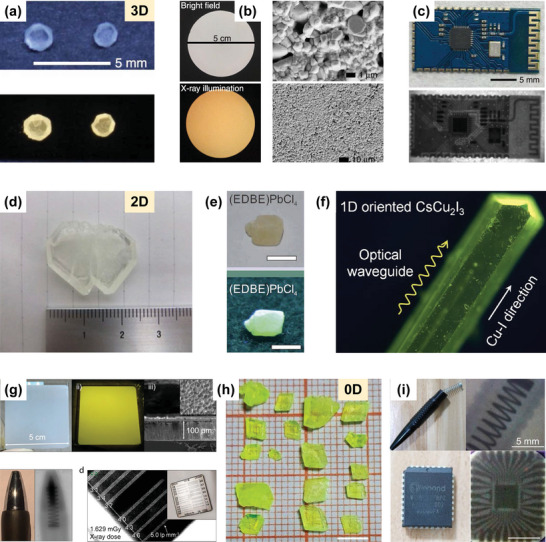
HP SCs with the structure change from 3D to 2D to 1D and to 0D for X‐ray detection. a) Pictures of 3D Cs_2_Ag_0.6_Na_0.4_In_0.85_Bi_0.15_Cl_6_ SCs under daylight and UV light excitation. b) Pictures and SEM images of the Cs_2_Ag_0.6_Na_0.4_In_0.85_Bi_0.15_Cl_6_ polycrystalline wafer. c) Picture and X‐ray image of a circuit board. Reproduced with permission.^[^ ^186^ ^]^ Copyright 2020, Springer Nature. d) Picture of a 2D (C_6_H_5_CH_2_CH_2_NH_3_)_2_PbBr_4_ SC. Reproduced with permission.^[^ ^189^ ^]^ Copyright 2017, Springer Nature. e) Pictures of a 2D (EDBE)PbCl_4_ SC under day light and ultraviolet lamp excitation. Reproduced with permission.^[^ ^16^ ^]^ Copyright 2016, Springer Nature. f) Picture of a 1D CsCu_2_I_3_ SC. g) Pictures, photoluminescent and SEM image of the 1D CsCu_2_I_3_ polycrystalline thin film, the X‐ray imaging also inserted. Reproduced with permission.^[^ ^205^ ^]^ Copyright 2021, American Chemical Society. h) Picture of the 0D Cs_4_PbBr_6‐x_Cl_x_ SCs. i) The X‐ray imaging based on the Cs_4_PbBr_6‐_ *_x_* Cl*_x_* SCs. Reproduced with permission.^[^ ^192^ ^]^ Copyright 2021, American Chemical Society.

HP SC thin films show great potential for optoelectronic applications. In particular, large‐area HP SC thin film with high LY is expected to be used as scintillator screen in X‐ray imaging. For scintillators, Conventional 3D HP SCs do not have sufficient scintillation performance at room temperature, which is due to the relatively low exciton binding energy.^[^ ^184^, ^185^ ^]^ Therefore, in order to increase the scintillator performance, Yang's group prepared the Bi^3+^ doped multicomponent double‐perovskite Cs_2_Ag_0.6_Na_0.4_In_0.85_Bi_0.15_Cl_6_ SC (**Figure** **28**a), which shows light yield of 39 000 ± 7000 photons MeV^−1^. higher were prepared by Yang's group for X‐ray detection.^[^ ^186^ ^]^ Based on the powder ground with the SC, they tested the potential for X‐ray imaging application (Figure 28b,c).

Since the 1990s 2D perovskite has been proposed and studied.^[^ ^187^ ^]^ Asai et al. first recorded the 2.0 MeV proton bombardment of 2D (C_6_H_13_NH_3_)_2_PbI_4_ thin films in 2002.^[^ ^46^ ^]^ At room temperature, both (C_6_H_13_NH_3_)_2_PbI_4_ and (C_3_H_7_NH_3_)_2_PbBr_4_ HP decay faster than many standard scintillators. Unfortunately, the thickness of the 2D crystals is <0.3 mm. It is worth noting that thickness is crucial in detecting high‐energy radiation. To obtain thicker 2D crystals, Koshimizu et al. selected aromatic ammonium to increase its thickness to over 1 mm. They prepared 5 mm × 6 mm × 1 mm SCs of (C_6_H_5_CH_2_CH_2_NH_3_) _2_PbBr_4_.^[^ ^188^ ^]^ This SC showed high LY of 10 000 photons MeV^−1^ under X‐rays. Recently, Kawano et al. reported an antisolvent diffusion method to synthesize large (C_6_H_5_CH_2_CH_2_NH_3_)_2_PbBr_4_ crystals with size of 17 mm × 23 mm × 4 mm (Figure 28d),^[^ ^189^ ^]^ which have excellent scintillation characteristics and show high LY of 14000 photon MeV^−1^ and short response time of 11 ns under X‐rays.^[^ ^189^ ^]^ In fact, the scintillation properties of HP SCs with similar structure were already investigated in 2016 (Figure 28e).^[^ ^16^ ^]^ In order to increase the structure distortion and optical yield, Horimoto et al. synthesized [n‐FC_6_H_4_(CH_2_)_2_NH_3_]_2_PbBr_4_ {*n*‐FPhe = FC_6_H_4_(CH_2_)_2_} (*n* = 2, 3, 4) crystals.^[^ ^9^ ^]^ However, under X‐rays, the optical yield is ≈3300 for 2‐FPhe, 12 000 for 3‐FPhe, and 8300 photon MeV^−1^ for 4‐FPhe, which is even lower than for the (C_6_H_5_CH_2_CH_2_NH_3_)_2_PbBr_4_ crystal.

The 2D HP has a robust quantum confinement effect and thus shows a large exciton binding energy that is ≈4× larger than for 3D perovskite.^[^ ^190^ ^]^ Due to the large exciton binding energy successfully inhibiting the thermal quenching effect, the 2D HP SC shows a high LY over 10 000 photons MeV^−1^ at room temperature.^[^ ^9^ ^]^ In addition, 2D HP has a faster radiation lifetime and better stability due to the special quantum‐well structure. These benefits make the 2D HP an excellent scintillator.^[^ ^191^ ^]^ At present, the widely used strategies for 2D crystal growth are the antisolvent diffusion method and slow solvent evaporation method, which often takes more than 2 weeks. In addition, the crystal easily grows into irregularly shaped particles and boundaries. Therefore, further research is needed to develop an efficient method to grow large‐scale high‐quality 2D crystals with good morphology.

In addition to 3D and 2D HP SCs, 1D SCs have also been successfully prepared for X‐ray detection. For example, Tang et al., prepared CsCu_2_I_3_ SCs first and studied their scintillation properties in X‐ray scintillator detector (Figure 28f).^[^ ^205^ ^]^ Due to the benefits including 1D crystal structure, the congruent‐melting feature, and the highly matching to spectral response of some universal photodetectors, such material shows high application potential in light‐emitting devices. Therefore, large area oriented nanorod structure polycrystalline films are prepared using the 1D CsCu_2_I_3_ SCs, and achieved X‐ray imaging with spatial resolution as high as 7.5 lp mm^−1^(Figure 28g). Furthermore, 0D structure HP Cs_4_PbBr_6‐_ *_x_* Cl*_x_* SCs have been also successfully grown and used in X‐ray detection and imaging studies (Figure 28h,i).^[^ ^192^ ^]^ The reported results prove that HP SC with low dimensional structure has better scintillation performance than the HP SC with 3D structure.

Despite the HP SCs with 3D, 2D, 1D, and 0D structures have been successfully grown for X‐ray scintillation detection, however, up to date, none of them have been directly used for X‐ray detection and imaging. The main reasons are as follows: 1) the size of above SC is limited, so it cannot be directly used for X‐ray imaging; 2) the quality and uniformity of the SCs is not enough; 3) the light yield of most reported HP SCs is still limited, which cannot meet the needs of high‐resolution X‐ray imaging.

### 5.3 HP Powder Scintillators

Halide double perovskite has recently become a green candidate for photoelectric devices, such as LEDs and X‐ray detectors. Zhu et al. reported some double‐perovskite Cs_2_Ag_0.6_Na_0.4_In_1‐y_Bi_y_Cl_6_ materials as scintillators.^[^ ^186^ ^]^ In this work, the X‐ray absorption coefficient, LY and light decay time were controlled to maximize the scintillator performance by adjusting the fraction of Bi^3+^ ions. As a result, the light production rate of Cs_2_Ag_0.6_Na_0.4_In_0.85_Bi_0.15_Cl_6_ achieved 39 000 ± 7000 photons MeV^−1^, which is far higher than the light production rate of CsPbBr_3_ (21 000 photons MeV^−1^) reported previously. For the scintillator, a large emission wavelength shift between the radiation luminescence (RL) and the absorption spectrum benefits from the self‐trapped excitons (STEs) effect and produces a negligible self‐absorption. Due to the high LY and speed of light attenuation, the scintillator can be used at a very low dose of ≈1 µGy_air_ to obtain static X‐ray images and operation at 47.2 µGy_air_ s^−1^ can realize dynamic X‐ray images without the ghost effect.^[^ ^186^ ^]^ Furthermore, after heating at 85 °C for 50 h and continuous irradiation under X‐ray in ambient air for 50 h, the scintillation properties remained basically unchanged in terms of both RL intensity and X‐ray imaging. More performance comparison of perovskite and other scintillators for X‐ray detection are summarized in **Table** **3**.

**Table 3:** Performance comparison of perovskite‐based and other scintillators for X‐ray detections

Materials	X‐ray tube voltage [kV]	Light yield [photons MeV^−1^]	Spatial resolution [lp mm^−1^]	Detection limit [nGy s^−1^]	Refs.
CsPbBr_3_ nanocrystals	‐	21 000	‐	‐	[193]
Cs_4_PbBr_6‐x_Cl_x_ SCs		>120 000			[16]
(EDBE)PbCl_4_		9000			[16]
(C_6_H_13_NH_3_)_2_PbI_4_					[46]
CsPbBr_3_ SC	55	50 000	‐	‐	[194]
MAPbI_3_ SC	45	<1000	‐	‐	[16]
MAPbBr_3_ SC	‐	<1000	‐	‐	[10]
PPO + CsPbBr_3_ NCs	70	≈530	3.5	3.74 × 10^7^	[179]
CsPbBr_3_@Cs_4_PbBr_6_ powders	‐	6000	‐	‐	[178]
CsPbBr_3_@Cs_4_PbBr_6_ nanocrystals	‐	64 000	‐	‐	[182]
(C_6_H_5_C_2_H_4_NH_3_)_2_PbBr_4_ SC	30	14 000	‐	‐	[189]
Li‐doped (PEA)_2_PbBr_4_ SC		11 000			
(C_6_H_5_C_2_H_4_NH_3_)_2_PbBr_4_ SC	50	10 000	‐	‐	[191]
Cs_2_Ag_0.6_Na_0.4_In_0.85_Bi_0.15_Cl_6_ SC		≈39 000		47 200	[186]
(C_6_H_5_C_2_H_4_NH_3_)_2_Pb_0.75_Ba_0.25_Br_4_SC	‐	19 000	‐	‐	[196]
(C_6_H_5_C_2_H_4_NH_3_)_2_Pb_1−𝑥_Sr_𝑥_Br_4_	40	19 700	‐	‐	[197]
Cs_4_SrI_6_:Eu	662	62 300	‐	‐	[168]
Cs_4_CaI_6_:Eu	662	51 800	‐	‐	[168]
Rb_2_CuCl_3_	50	16 600	‐	88.5	[198]
Rb_2_CuBr_3_ SC	50	91 056	‐	121.5	[199]
Cs_2_TeI_6_ films	662	32 000	‐	‐	[122]
Cs_2_HfCl_6_	662	54 000	‐	‐	[122]
Rb_2_CuBr_3_		97 000			[200]
K_2_CuBr_3_	50	23 806		132.8	[201]
CsCu_2_I_3_ polycrystalline	70	‐	7.5	1.629 × 10^6^	[16]
Cs_4_EuBr_6_ SC	35	78 000	‐	‐	[202]
Cu_3_Cu_2_I_5_ Powder−PDMS film		‐	6.8		[203]
Cs_3_Cu_2_I_5_‐PDMS film		‐	17	48.6	[204]
CsCu_2_I_3_	30		7.5	10–130	[205]
CsPbBr3 PMMA film	30	‐	12.5	120	[206]
CsPbBr_3_ QD‐poly(3‐hexylthiophene) (P_3_HT)		‐	5		[207]
K^+^‐Cs_3_Cu_2_Cl_5_ Nanosheets		≈85 000	10.7	16	[208]
Mn (II) ‐BA_2_PbBr_4_		85	20	16	[209]
LiYbF_4_:Tb NCs	50		20	360	[210]
Tb^3+^‐doped Ba_2_LaF_7_ NC s					[211]
[4‐FC_6_H_4_(CH_2_)_2_NH_3_]_2_PbBr_4_ crystals		8300			[9]
Cs_4_PbBr_6_−xCl_x_ SCs	50			3.45 × 10^6^	[192]
Cs_2_Ag_0.6_Na_0.4_In_0.85_Bi_0.15_Cl_6_			4.3		[192]
Cs_2_NaTbCl_6_	30	46 600		3.45 × 10^6^	[212]
Cs_2_NaEuCl_6_	30	1250		3.45 × 10^6^	[212]
CsI:Tl	60	52 000			[213]
Gd_2_O_2_S		60 000			[212]
BGO	‐	≈8500	‐	‐	[214]
CaF_2_	‐	24 000	‐	‐	[193]
CsPbCI_3_ SC	50	330	‐	‐	[215]
CeBr_3_	‐	60 000	‐	‐	[216]

## 6 Flexible HP X‐Ray Detectors

Flexible photodetectors based on HPs have become a new type of optoelectronic device^[^ ^217^ ^]^ due to their tunable optoelectronic characteristics, excellent photosensitivity, remarkable mechanical flexibility and relatively low‐temperature solution processability.^[^ ^206^ ^]^ Flexible X‐ray detectors show great potential in medicine, industry, national security and scientific research.^[^ ^218^ ^]^ Since they can bend to accommodate non‐flat surfaces or observe objects in a limited space, rather than externally imaging objects by filtering X‐rays from non‐target parts of objects, flexible X‐ray detectors can produce clearer images using fewer X‐rays than rigid detectors. In addition, flexible X‐ray detectors can operate in non‐uniform X‐ray fields. The following sections will discuss the progress of direct and indirect flexible photodetectors.

### 6.1 Flexible Direct‐Type X‐Ray Detectors

**Figure 29:**
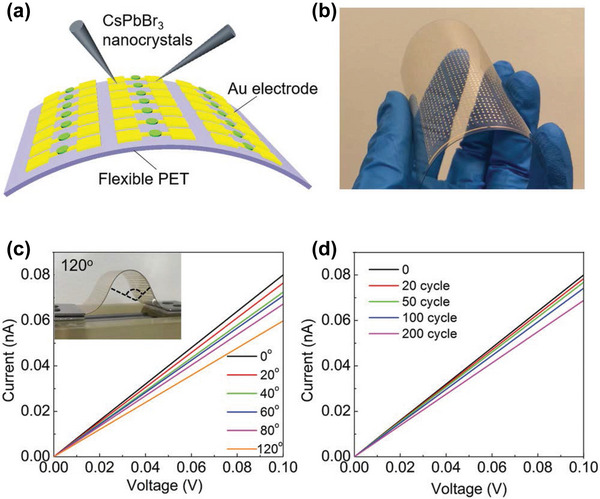
Flexible HP direct‐type X‐ray detectors. a) Illustration of the flexible CsPbBr_3_ QD X‐ray detector. b) Photograph of the CsPbBr_3_ QD X‐ray detector. c) *I–V* curves of the detector arrays when bending at different angles. d) *I—V* curves of the flexible detector arrays after bending for 20, 50, 100, and 200 cycles. Reproduced with permission.^[^ ^6^ ^]^ Copyright 2019, John Wiley and Sons.

Perovskite semiconductors are promising candidates of active materials for flexible X‐ray detectors because of their cost‐effectiveness and low‐temperature processability. In 2019, Liu et al. fabricated the first flexible HP X‐ray detector arrays by printing CsPbBr_3_ films onto PET substrates (**Figure** **29**a,b).^[^ ^6^ ^]^ Flexible devices can be easily bent at different angles. The current of the CsPbBr_3_ X‐ray detector array under X‐ray radiation at different bending angles (7.33 mGy_air_ s^−1^) varies with voltage (0–1 V) in shown in Figure 29c,d. The strain generated by 200 bending cycles reduced the current by only 12%. After three months in the environment, it still worked well. However, under the same dose rate of 7.33 mGy_air_ s^−1^ and biased at 0.1 V, a sensitivity of 17.7 µC Gy^−1^ cm^−2^ was obtained, which is 83 µC Gy^−1^ cm^−2^ lower than that of the rigid detector. This may be related to the lower film thickness, which generates fewer carriers in the flexible detector.

**Figure 30:**
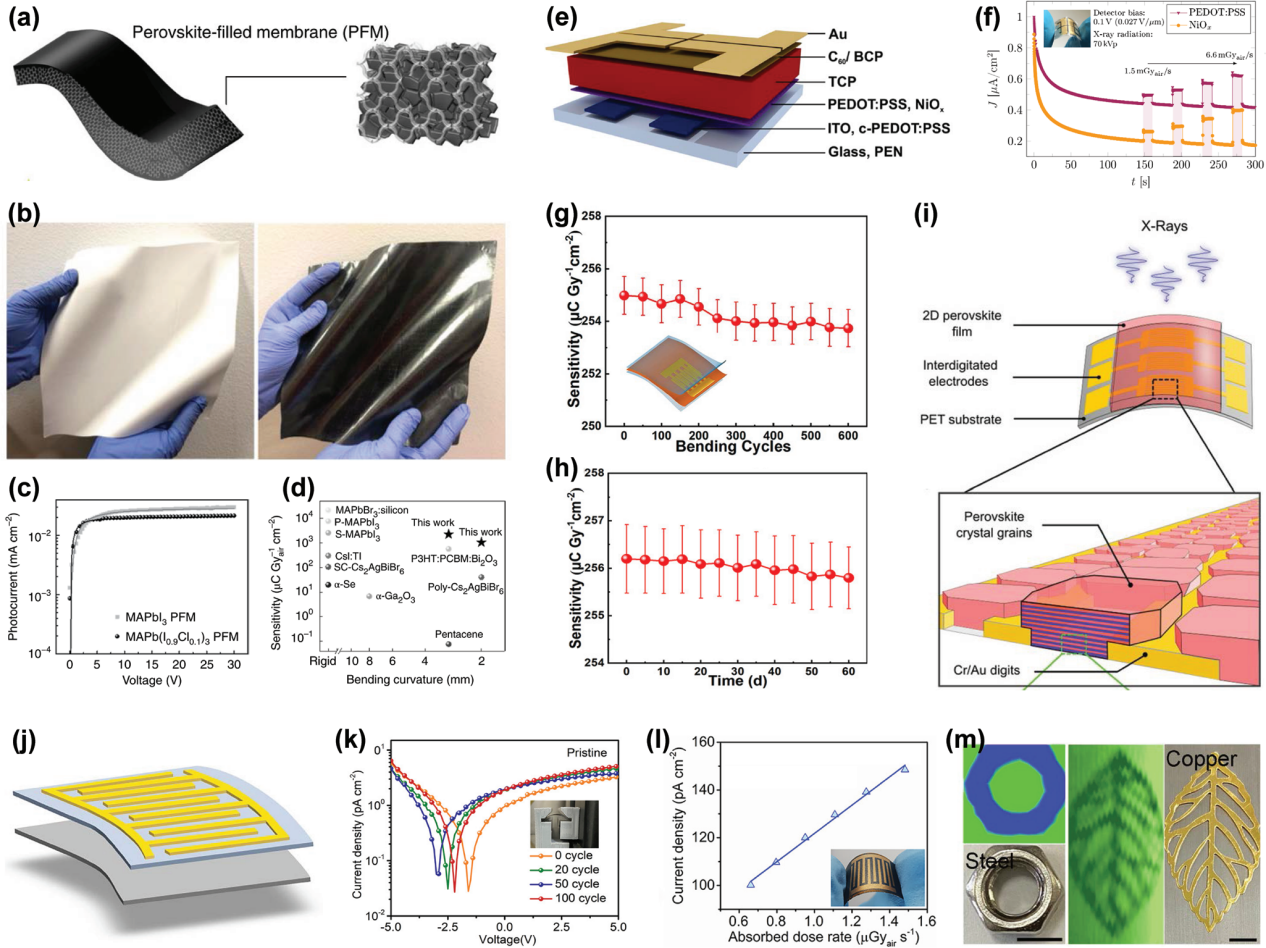
Flexible HP direct‐type X‐ray detectors. a) Schematic diagram of the perovskite‐filled nylon membrane. b) Photographs of a flexible nylon membrane with area of 400 cm^2^. c) Photoconductivity of the MAPbI_3_ and MAPb(I_0.9_Cl_0.1_)_3_PFM device. d) Comparison of the measured X‐ray detection sensitivity of flexible X‐ray detectors. Reproduced with permission.^[^ ^85^ ^]^ Copyright 2020, Springer Nature. e) X‐ray detector architecture and layer stack. f) X‐ray response of flexible triple‐cation perovskite thin‐film X‐ray detector. Reproduced with permission.^[^ ^86^ ^]^ Copyright 2020, American Chemical Society. g) Sensitivity of the Au/Cs_4_PbI_6_/Au detector after bending for 600 cycles at an angle of 90°. h) X‐ray response sensitivity versus the storage time in air. Reproduced with permission.^[^ ^121^ ^]^ Copyright 2021, American Chemical Society. i) Schematic of a 2‐terminal X‐ray detector fabricated on PET substrate. Reproduced with permission.^[^ ^219^ ^]^ Copyright 2022, John Wiley and Sons. j) Sketches of Cs_2_TeI_6_ detector structures based on flexible substrate. k) *J–V* curves of the flexible detector after bending for different numbers of cycles. l) Xray current density dependence on the X‐ray dose rate. m) X‐ray images of an M6 nut and a copper leaf using the Cs_2_TeI_6_ film detector. Reproduced with permission.^[^ ^122^ ^]^ Copyright 2021, American Chemical Society.

HP SCs have been widely used in X‐ray detection studies, but the brittleness of SCs limits their construction and the integration on readout circuits. Metal HP has the potential as a flexible X‐ray detector because of its softness. Based on the composition and crystallinity, the young's modulus of HP SCs is between 2.51 and 20 GPa, which is equivalent to the value of polymers (0.92–4.3 Gpa);^[^ ^85^ ^]^ however, HP SCs are still too brittle to be used in flexible detectors. In addition, the submillimeter‐thick polycrystalline perovskite film does not have high mechanical strength to fracture along the grain boundaries. Recently, Huang's group prepared a large area (400 cm^2^) flexible X‐ray detector using nylon film filled with MAPbI_2.7_Cl_0.3_ (**Figure** **30**a,b).^[^ ^85^ ^]^ The detector fabricated on the thin film shows a *µτ* product of 1.5 × 10^−3^ cm^2^ V^−1^ and realized a high sensitivity of 8689 µC Gy_air_^−1^ cm^−2^. It should be noted that the detector did not lose efficiency even after it was bent to a radius of 2 mm (Figure 30c,d). Mescher et al. presented a flexible X‐ray detector by replacing the poly (3,4‐dioxythiophene)/poly (styrenesulfonate) (PEDOT:PSS) with NiO*_x_* as the transport layer (Figure 30e) and obtained a sensitivity of 59.9 µC Gy_air_^−1^ cm^−2^ under 70‐kVp X‐rays and at a low voltage of only 0.1 V (Figure 30f).^[^ ^86^ ^]^ Recently, Xu's group designed a polymer‐encapsulated Au/Cs_4_PbI_6_/Au flexible detector that achieved high sensitivity and air stability X‐ray detection.^[^ ^121^ ^]^ The detector displayed a sensitivity of 256.20 µC Gy^−1^ cm^−2^ under a 30‐keV X‐ray source. Due to the protection of the polymer film, the detector was stable in air and after enduring flexing. After 60 days in air or 600 bending cycles, the sensitivity remained almost the same (Figure 30g,h).

2D perovskite is often crystallized to form a self‐assembled quantum well structure and has exciting characteristics such as low defect‐state density. Fraboni et al. proposed a solid‐state ionizing radiation detector based on 2D HP PEA_2_PbBr_4_ (PEA = C_6_H_5_C_2_H_4_NH_3_^+^).^[^ ^219^ ^]^ A scalable technology was utilized to deposit the detector from solution and it was directly integrated onto a pre‐etched flexible substrate. It showed crystal‐like behavior in the form of a HP thin‐film, which was proved by fast and good emission performance under UV light (Figure 30i). In addition, the measurement results showed an abnormally stable response under continuous irradiation and bias, which used to evaluate the robustness of the materials and the close electrical contact between the electrodes and HPs. Interesting research into flexible X‐ray detectors based on different materials and densities is ongoing. Xu et al. reported a flexible X‐ray detector made of lead‐free Cs_2_TeI_6_ film on polyimide (PI) substrate by low‐temperature electrospraying (Figure 30j).^[^ ^122^ ^]^ The resistivity remained at 10^11^ Ω cm during 100 bending cycles at a bending radius of 10 mm (Figure 30k). The flexible Cs_2_TeI_6_ detector shows better X‐ray response stability than the rigid SnO_2_:F glass (FTO) detector, because the flexible substrate has better crystallization characteristics and growth stress release characteristics. The X‐ray sensitivity was 76.27 µC Gy_air_^−1^ cm^−2^, and satisfactory X‐ray imaging was obtained (Figure 30l,m).

### 6.2 Flexible Indirect‐Type X‐Ray Detectors

**Figure 31:**
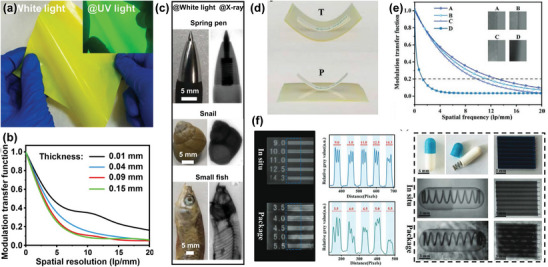
Flexible HP indirect‐type X‐ray detectors. a) Photograph of the CsPbBr_3_@PMMA films under the illumination of UV light. b) The modulation transfer function (MTF) of CsPbBr_3_@PMMA films with different thickness (0.01, 0.04, 0.09, 0.15 mm). c) X‐ray images obtained from the CsPbBr_3_@PMMA films. Reproduced with permission.^[^ ^220^ ^]^ Copyright 2022, John Wiley and Sons. d) Photograph of the CsPbBr_3_ polymer‐ceramics. e) The MTF of the CsPbBr_3_ polymer‐ceramics. f) X‐ray images and photograph of the standard X‐ray resolution pattern plate and the encapsulated spring. Reproduced with permission.^[^ ^206^ ^]^ Copyright 2022, John Wiley and Sons.

Recently, HP nanocrystals are incorporated into the polymer matrix to prepare flexible HP thin films that can be prepared to any expected shape. Wang et al. prepared large‐sized, flexible, and stable CsPbBr_3_@polymethyl methacrylate composite film (**Figure** **31**a).^[^ ^220^ ^]^ After bending it 2000 times and storing it in water for >2520 h, the composite film retained 94% and 81% of the pristine PL intensity, respectively. Additionally, it shows a detection limit as low as 40.1 nGy_air_ s^−1^, spatial resolution as high as 8.0 lp mm^−1^, and good X‐ray irradiation tolerance (108 h), as shown in Figure 31b,c. Based on high viscosity, Xu et al. designed perovskite “polymer ceramic” scintillators for flexible and refreshable X‐ray imaging.^[^ ^206^ ^]^ The scintillator detector has a detection limit of 120 nGy s^−1^ and spatial resolution of 12.5 lp mm^−1^ (Figure 31d–f).

**Figure 32:**
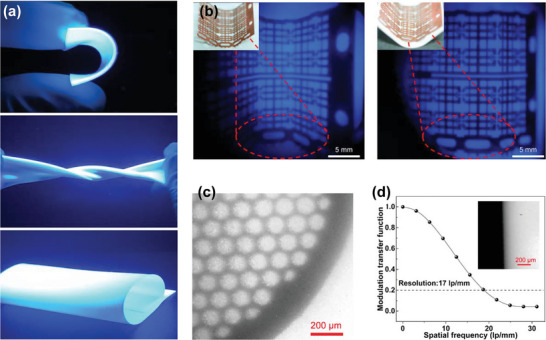
Flexible Cu‐base HP indirect‐type X‐ray detectors. a) The Cs_3_Cu_2_I_5_‐polydimethylsiloxane film (CCI‐P) under UV excitation: bending, twisting, and folding. b,c) X‐ray images of the flexible copper grid obtained using a 50‐µm CCI‐P film. d) MTF of the Cs_3_Cu_2_I_5_‐polydimethylsiloxane film. Reproduced with permission.^[^ ^204^ ^]^ Copyright 2022, American Chemical Society.

Cu‐base halides with high PLQY and large Stokes shift because of their low electronic dimension can produce scintillators without reabsorption. Zhou et al. prepared a flexible Cu_3_Cu_2_I_5_‐PDMS film with high scintillation yield by ball milling Cu_3_Cu_2_I_5_ powder.^[^ ^204^ ^]^ The PLQY of Cu_3_Cu_2_I_5_‐PDMS thin film (90%) exceeded that of Cu_3_Cu_2_I_5_ powder (74.5%), which may be due to the passivation effect of PDMS leading to a longer carrier lifetime (**Figure** **32**a). Therefore, the Cu_3_Cu_2_I_5_‐PDMS thin film realized clear X‐ray imaging with a spatial resolution of 17 lp mm^−1@0.2^ MTF (Figure 32b,c). Additionally, Li et al. prepared a flexible copper halide film with an area of 400 cm^2^, which had excellent mechanical stability, good uniformity, ≈100% PLQY, water resistance and heat resistance.^[^ ^203^ ^]^ The self‐absorption‐free scintillators designed on these films showed excellent emission performance, with a low detection limit of 48.6 nGy s^−1^ and spatial resolution of 17 lp mm^−1^. Jin et al. prepared regionally controllable uniform and flexible zero‐dimensional potassium doped Cs_3_Cu_2_Cl_5_‐polystyrene thin films with excellent X‐ray sensitivity, photoluminescence quantum yield (70.23–81.39%), radiation luminescence intensity and stability.^[^ ^208^ ^]^ Furthermore, this flexible film has a low cost (US $2.8223 g^−1^), excellent response to 20–160 keV X‐rays with high spatial resolution of 5 lp mm^−1^, which is comparable to the commercial CsI:Tl wafers.

## 7 Challenges of HP X‐Ray Detection

There are several benefits of HPs for X‐ray detection and much has been achieved in the recent years; however, considering the practical application conditions and environments of X‐ray detectors, many figures of merit besides the detection performance should also be addressed. Therefore, many challenges of next‐generation X‐ray detectors based on HPs remain to be solved in the future, for example, the environmental stability of HP for direct detection, large‐scale preparation of HP for multi‐pixel integration, and scintillation stability of the scintillators.

### 7.1 Stability of HPs for Direct‐Type Detectors

Compared with the traditional inorganic semiconductors such as Si, the long‐term stability of HPs is still a key problem. For inorganic perovskite, although its thermodynamic stability is better than that of hybrid perovskite, there are still phase transition problems. Therefore, in order to be further applied in optoelectronic devices, the stability problem must be overcome. Although the stability of HPs under ambient environmental conditions has been widely studied, their stability when operating in high‐energy radiation fields needs further evaluation. Ion migration (also known as hysteresis) is a well‐known problem in HP detectors under high‐energy radiation, because its response and noise are both directly related to it. Therefore, unrestrained ion migration will not only lead to obvious baseline drift, affect signal collecting and cause overflow error, but also increase the dark current and scattering noise.^[^ ^165^, ^221^ ^]^ Hysteresis caused by ion migration not only harms the performance but also degrades long‐term stability of the device. While the defects are the main channels of ion migration, therefore, it is advisable to reduce the density of bulk defects in HPs as much as possible. The main strategies for improving perovskite quality and reducing defects will discuss in the following section.

### 7.2 Large‐Area Growth of HP for Direct‐Type Detectors

Imaging applications require large‐area semiconductors that are equal to the size of the target objects. However, for direct X‐ray detectors, the lack of materials with strong X‐ray attenuation, excellent charge transfer characteristics and, especially, large‐area production compatibility is still a problem. As an excellent scintillator, 2D HP exhibits high LY exceeding 10 000 photons MeV^−1^ even at room temperature. In addition, due to the quantum‐well structure, it has a shorter radiation lifetime and higher environmental stability. However, the challenge is to grow large‐size high‐quality 2D HP SC with good morphology by a solution method. Although perovskite thin films can be obtained with large area and flexible properties, there are still great problems with their quality and thickness. Therefore, it is challenging to prepare perovskite in a large‐area form with flexibility and considerable thickness.

### 7.3 Scintillation Stability of Scintillators

3D perovskite has outstanding photovoltaic characteristics, for example, the long carrier diffusion distance and low exciton binding energy. However, the phase transition and instability of these 3D perovskites hinder their practical application in the ambient environment. In order to avoid these drawbacks of 3D perovskites, low‐dimensional HPs have been established. Compared with 3D, 2D, and 1D perovskites, 0D perovskite has the best stability and is more conducive to practical applications. 3D perovskite usually has a small Stokes shift, while 0D perovskite has a large Stokes shift, which makes it more useful as an X‐ray scintillator. However, there is still a lack of further research on the properties, synthesis, and applications of 0D perovskite SC.^[^ ^4^ ^]^

## 8 Perspective

### 8.1 Develop Protection Strategies for HPs and Corresponding Devices

#### 8.1.1 Surface Passivation

According to the reported results, ion migration mainly occurs at the grain boundaries through defect sites. The introduction of a passivation layer or interface design can minimize the impact of ion migration. Surface passivation and surface ligand engineering can reduce interface defects and promote carrier transfer among nanocrystals (NCs). Since the surface modification of NCs will affect the charge transfer efficiency in the thin film, the surface passivation method also needs to consider the carrier transfer at the interface. For example, in the 0D perovskite‐2D MoS_2_ system, the surface ligand density control strategy that balances the surface passivation of quantum dots and the efficiency of interface charge extraction/injection improves the interface contact between QDs and MoS_2_, thereby improving the interface carrier transfer efficiency.^[^ ^222^ ^]^ In addition, modifying the unstable ligand with external insulation of nanocarbon is also conducive to carrier transfer.

#### 8.1.2 Develop Effective Encapsulation Strategies

HP‐based X‐ray scintillators still have poor stability, which hinders their further application in wearable devices, non‐planar objects, and narrow‐space imaging targets. In order to meet the potential demand of commercial X‐ray imaging, scintillators with large size, stability, flexibility, and high performance are being researched and developed. Generally, this packaging structure is used for thin‐film packaging of flexible devices. Polymer nanomaterials with reasonably high dielectric constant and high‐throughput production are good candidates for packaging metal HP‐based FPDs, including poly(vinyl alcohol‐*co*‐polymer)(PVA), Surlyn, polyethylene terephthalate (PET), polytetrafluoroethylene (PTFE), polycarbonate (PC), poly(*p*‐chloro‐xylene) (parylene‐c), and poly(ethylene vinyl alcohol) (EVOH). Because polymer film meets the requirement for transmission of X‐rays, it is suitable for encapsulation of X‐ray detectors. Polymers like PET, PTFE, PMMA, etc. can be used for encapsulation, and the well‐developed processes such as chemical vapor deposition (CVD), plasma enhanced chemical vapor deposition (PECVD), physical vapor deposition (PVD), atomic layer deposition (ALD), etc. can be used to prepare Al_2_O_3_ or SiO_2_ barrier layers. In addition, metal foil can also be used for packaging. However, the water–vapor transmission of polymer encapsulation, TFE and metal foil must be also considered with regards to detection performance.

### 8.2 Process Optimization During Crystallization of HPs

#### 8.2.1 Optimization for Single‐Crystal Growth

An imperfect SC can affect the LY by capturing photoexcited electrons. More effort is needed to grow high‐quality SCs with clear morphology for efficient device performance. Recently, lead‐based HPs have been developed for sensitive X‐ray detection due to their large density, strong X‐ray photo absorption, long carrier lifetime, and large mobility. In addition, the lead HPs can be produced as large‐size SC using various processes at low temperature. Furthermore, the combination of optimizing single‐crystal growth, designing new structures, and synthesis of lead‐free perovskite materials needs to be further developed.

#### 8.2.2 New Strategies for Large‐Area HPs

Although a variety of solution‐growth processes have been explored, the large‐area production of high‐pressure single crystals is more challenging. Flexible photodetectors have become a new category of optoelectronic devices with large‐area compatibility, light weight, low cost, and adaptability to flexible soft surfaces. The preparation techniques of large‐area perovskite films include screen printing, coating, pressing, and injection.

### 8.3 Design and Optimization of New HP Perovskites

#### 8.3.1 Ion Doping Engineering

Doping technology is widely used in the improvement of perovskite scintillators, and the Stokes shift can be further extended by doping technology. For the doping strategy, the doped transition metal ions can become recombination centers to generate scintillation emission of wavelengths with the energy matching the transition gap. Furthermore, the intrinsic traps in the band gap also will influence the scintillation performance after doping. In the past decade, the changes of electronic properties due to the intrinsic defects in perovskite have been well studied by DFT, which provides detailed theoretical guidance for the future research of defect‐adjusted scintillation.

#### 8.3.2 Compositional Engineering

Although some current studies mainly focus on improving the operational stability of photodetection by using a buffer layer through interface engineering, controlling the perovskite crystal morphology and passivating perovskite surface defects, and, more importantly, developing highly chemically stable HPs are conducive to fundamentally solving the stability problem of perovskite‐based X‐ray detectors. As demonstrated in previous works, composition engineering by mixing CH_3_NH_3_^+^, CH (NH_2_)_2_^+^, Cs^+^ cations and I^−^, Br^−^, Cl^−^ anions in HPs may be an effective method to realize higher stability than is achieved with pure HPs. In order to improve both the performance and the stability, it is very attractive to introduce collaborative component engineering, that is, the A‐site alloy reduces the trap density, and the B‐site doping releases the micro strain caused by the A‐site alloy. For example, the mixed cation/halide Cs*_X_* FA_1‐_ *_X_* Pb(I_1‐_ *_y_* Br*_y_*)_3_ and Cs_2_AgBiBr_6_ SCs show superior performance and good stability in X‐ray detection.

#### 8.3.3 Control of Crystalline Orientation

In 2D HP, different crystal planes show different carrier transport characteristics. Therefore, we can improve the sensitivity of X‐ray detection by controlling the superior crystallization orientations. In addition, the oriented perovskites have good carrier collection and inhibition of ion migration under a large applied bias in the off‐plane direction, which is beneficial to further decrease the detection limit. In general, the rapid pressing process is a simple and effective method to obtain large‐area 2D wafers with uniform orientation, and they have demonstrated high X‐ray detection sensitivity and better operational stability than the conventional 2D perovskite SCs.

Currently, the use of HPs in X‐ray detectors is still in its infancy, and scintillators used in indirect conversion are being manufactured on the laboratory scale. In the future, efforts to further understand the crystal orientation effect, surface and interface passivation, ion doping, and composition tuning are the still the main directions to improve the performance of HP X‐ray detectors. Repeatability, large‐size preparation and environmental friendliness of HPs are expected to be realized in next decades, finally leading to commercialization.

## Conflict of Interest

The authors declare no conflict of interest.
